# Clinical and radiological features of lung disorders related to connective-tissue diseases: a pictorial essay

**DOI:** 10.1186/s13244-022-01243-2

**Published:** 2022-06-29

**Authors:** Stefano Palmucci, Federica Galioto, Giulia Fazio, Agata Ferlito, Giovanna Cancemi, Alessia Di Mari, Gianluca Sambataro, Domenico Sambataro, Giovanni Zanframundo, Letizia Antonella Mauro, Pietro Valerio Foti, Carlo Vancheri, Antonio Basile

**Affiliations:** 1 Radiology Unit 1, Department of Medical Surgical Sciences and Advanced Technologies “GF Ingrassia”, University Hospital Policlinico “G. Rodolico-San Marco”, 95123 Catania, Italy; 2 Department of Clinical and Experimental Medicine, Regional Referral Centre for Rare Lung Diseases, A. O. U. Policlinico “G. Rodolico - San Marco”, University of Catania, Catania, Italy; 3 Artroreuma S.R.L, Rheumatology Outpatient Clinic Associated with the National Health System, Mascalucia, Catania, Italy; 4 Rheumatology Division, Department of Internal Medicine and Therapeutics, University of Pavia and IRCCS Policlinico S. Matteo Foundation, Pavia, Italy; 5 Radiology Unit 1, University Hospital Policlinico “G. Rodolico-San Marco”, 95123 Catania, Italy

**Keywords:** Connective tissue disease, Multidetector computed tomography, Lung disease (Interstitial), Autoimmune diseases, Pulmonary fibrosis

## Abstract

Connective tissue diseases (CTDs) include a spectrum of disorders that affect the connective tissue of the human body; they include autoimmune disorders characterized by immune-mediated chronic inflammation and the development of fibrosis. Lung involvement can be misdiagnosed, since pulmonary alterations preceded osteo-articular manifestations only in 20% of cases and they have no clear clinical findings in the early phases. All pulmonary structures may be interested: pulmonary interstitium, airways, pleura and respiratory muscles. Among these autoimmune disorders, rheumatoid arthritis (RA) is characterized by usual interstitial pneumonia (UIP), pulmonary nodules and airway disease with air-trapping, whereas non-specific interstitial pneumonia (NSIP), pulmonary hypertension and esophageal dilatation are frequently revealed in systemic sclerosis (SSc). NSIP and organizing pneumonia (OP) may be found in patients having polymyositis (PM) and dermatomyositis (DM); in some cases, perilobular consolidations and reverse halo-sign areas may be observed. Systemic lupus erythematosus (SLE) is characterized by serositis, acute lupus pneumonitis and alveolar hemorrhage. In the Sjögren syndrome (SS), the most frequent pattern encountered on HRCT images is represented by NSIP; UIP and lymphocytic interstitial pneumonia (LIP) are reported with a lower frequency. Finally, fibrotic NSIP may be the interstitial disease observed in patients having mixed connective tissue diseases (MCTD). This pictorial review therefore aims to provide clinical features and imaging findings associated with autoimmune CTDs, in order to help radiologists, pneumologists and rheumatologists in their diagnoses and management.

## Key points

- Pulmonary involvement may be observed in Connective tissue diseases (CTDs).
- On HRCT, pulmonary lesions may precede the onset of respiratory symptoms.
- Usual interstitial pneumonia (UIP), non-specific interstitial pneumonia (NSIP), and organizing pneumonia (OP) represent patterns of interstitial diseases in CTDs.
- Airway diseases and pleural effusions may be found in RA.
- Pulmonary hypertension is often depicted in scleroderma and systemic lupus erythematosus.

## Background

Connective tissue diseases (CTDs) include a spectrum of disorders that involve connective tissues of the human body; the target of these pathologies is represented by the extracellular matrix, which supports organs and is mainly composed of collagen and elastin. CTDs are “characterized by immune-mediated chronic inflammation often leading to tissue damage, collagen deposition and possible loss of function of the organ” [1]. They can be distinguished as heritable forms and autoimmune diseases. The latter have also been referred as systemic autoimmune diseases, which recognize genetic and environmental factors [2, 3]; they are mainly represented by rheumatoid arthritis (RA), systemic sclerosis (SSc), polymyositis (PM) and dermatomyositis (DM), systemic lupus erythematosus (SLE), Sjögren syndrome (SS) and mixed connective tissue diseases (MCTDs).

Pulmonary involvement can be observed in all these above-mentioned autoimmune diseases—often after the articular and skeletal symptoms and signs; however, an early identification of respiratory alterations is crucial for the prognosis, since lung involvement increases morbidity and mortality [4]. Multiple respiratory structures could be affected (pleura, parenchyma, airways, pulmonary vessels and thoracic muscles); more in detail, interstitial lung diseases (ILDs) may be encountered in almost all these autoimmune disorders, resembling different pulmonary patterns—such as usual interstitial pneumonia (UIP), non-specific interstitial pneumonia (NSIP), organizing pneumonia (OP). Moreover, in patients with CTDs and interstitial patterns observed on high-resolution computed tomography (HRCT), survival is better than that observed with regard to idiopathic pulmonary fibrosis (IPF) [5].

Recently, within the spectrum of ILDs, a subset of patients has shown a progressive course of pulmonary fibrotic disease—despite treatment; this ILD progression, characterized by an increased extent of fibrosis on HRCT and/or reduction of pulmonary function test values, has been defined as “ILD progressive fibrotic phenotype” [6, 7].

Moreover, a recent trial has registered the effect of antifibrotic therapy in slowing the progression of fibrosing ILDs, and this efficacy has been validated also for those associated with autoimmune diseases [8]. In view of these considerations, early recognition of pulmonary involvement by clinicians and radiologists is more favorable, to evaluate the possibility of treatment. Therefore, the aim of this pictorial review is to describe the main clinical and radiological patterns of lung involvement in autoimmune CTDs (Table 1), in order to increase radiologists’ familiarity with their pattern of lung presentation on imaging.

**Table 1:** Main serum markers, clinical features and radiological patterns of autoimmune lung CTDs

CTD	Serum markers	Clinical features	HRCT features
RA	Anti-CCP, RF	Erosive and symmetric inflammatory arthropathy, subcutaneous nodules, skin ulceration *Lung involvement:* exertional dyspnea non-productive cough, bibasilar inspiratory crackles (edema, cyanosis and signs of pulmonary hypertension)	UIP, NSIP patterns Airway disease with obliterative and follicular bronchiolitis Rheumatoid nodules
SSc	ACA Anti-Scl70, anti-RNA polymerase III	Skin thickening, telangiectasias, fingertip ulcers, gastrointestinal diseases, myocardial diseases *Lung involvement:* exertional dyspnoea, non-productive cough fatigue, “velcro” crackles	NSIP pattern, UIP pattern with straight-edge sign and/or “Four Corners” sign PH
IIM	Increased CK levels; positivity for MSAs/MAAs	Muscle weakness, systemic involvement and specific cutaneous manifestations (for DM heliotrope rash and Gottron papules) *Lung involvement:* (a) rapidly progressive dyspnea and respiratory failure. (b) insidious onset of dyspnoea	Hypoventilation and respiratory failure due to respiratory muscles involvement; aspiration pneumonia NSIP or OP pattern
SLE	ANA Anti dsDNA antibodies; anti-Sm; lupus anticoagulant Ro/SSA antibodies; hypocomplementemia	Rheumatological, dermatological and renal abnormalities *Lung involvement:* acute symptoms (dyspnoea, respiratory failure)—in cases of hemorrhage of ALP; dyspnea and cough in interstitial pneumonitis	Pleuritis, pleural effusions, pulmonary infections, chronic interstitial lung diseases (with NSIP pattern), ALP, DAH
pSS	ANA, anti-Ro60kD; anti-Ro52kD; anti-La	Xerostomia and xerophthalmia, focal lymphocytic sialadenitis *Lung involvement:* it could be asymptomatic In symptomatic cases, cough and dyspnea are often present	NSIP pattern LIP with diffuse interstitial and peribronchiolar infiltration of lymphoplasma cells OP pattern
MCTD	Anti-U1 RNP, anti-Ro52 antibodies	Patients are often asymptomatic Dysphagia and RP, diffuse hand edema (puffy hands)	NSIP, UIP; OP and DAD have been occasionally described

ACA, anticentromere antibodies; IIM, idiopathic inflammatory myopathy; ANA, antinuclear antibodies; MCTD, mixed connective tissue disease; MAA, myositis associated antibodies; MSAs, myositis specific antibodies; pSS, Primary Sjӧgren’s Syndrome; RA, rheumatoid arthritis; RF, rheumatoid factor; SLE, systemic lupus erythematosus; IIM, The term idiopathic inflammatory myopathy includes polymyositis, dermatomyositis, antisynthetase syndrome; MAA, this group of antibodies includes anti-Ro52kD, anti Pm/Scl 100 and 75Kd, anti-Ku, anti-RNP; MSA, this group of antibodies includes antisynthetase antibodies (anti-Jo1, PL7, PL12, EJ, OJ, Ks), anti-Mi2, anti SAE, anti-MDA5

## Main text

### Rheumatoid arthritis (RA)

RA is the most common connective tissue disease, with a prevalence of 1–2% in the general population; it commonly occurs in women, with a female to male ratio equal to 3:1 [7, 9]. This disease is characterized by erosive and symmetric inflammatory arthropathy, especially involving the distal joints. The evidence of early morning joint stiffness and pain—associated with positive rheumatoid factor (RF) and signs of arthritis—are considered typical clinical findings which may suggest the suspicion of disease. Highly specific for RA are the anti-cyclic citrullinated peptide (CCP) antibodies, which are usually related to the severe stage of joint disease [10].

Extra-articular manifestations include subcutaneous nodules, skin ulceration, pericarditis, splenomegaly, pericarditis, atherosclerotic artery disease and a wide spectrum of pulmonary manifestations [11].

#### Lung involvement

Pulmonary manifestations, which occur in up to 80% of RA patients, contribute significantly to an increase in morbidity. It may result directly from RA involvement, or may be a consequence of medical treatment with immune-modulating molecules, which increase the risk of opportunistic infections and/or episodes of drug toxicity [11].

Pulmonary lesions may precede articular symptoms in a relatively low percentage of cases (up to 20%), and are often detected within the first 5 years of disease [12]. Among CTDs, RA is characterized by heterogeneous lung involvement; all pulmonary structures—such as airways, pleura, lung vessels and parenchyma—may be interested. Interstitial pneumonitis and fibrosis, rheumatoid nodules, OP, bronchiectasis, obliterative bronchiolitis, follicular bronchiolitis, and pleural effusion are the most common manifestations encountered [13].

ILD is the most common pulmonary manifestation of RA, occurring in 10–20% of cases; it is frequently found in men aged between 50 and 60 years [7, 13]. The estimated prevalence of RA-ILD is heterogeneous: HRCT is more sensitive than other methods—such as pulmonary function tests (PFTs) and chest radiographs [14]. In addition, some published studies have shown that patients who underwent screening procedures based on CT examinations, in absence of respiratory symptoms, often exhibit radiological abnormalities referred as interstitial lung abnormalities (ILA).

There are some risk factors to development of ILD-RA: older age, male sex, smoking, longer disease duration, high level of RF and anti-CCP antibodies have been strongly associated with increased risk of disease [7, 12]. Namely, RF and anti-CCP antibodies also predict development of RA in asymptomatic or minimally symptomatic patients. They have been reported as the most important clinical features of an underlying CTD—in patients having isolated ILD and a history of smoking [10].

Other relevant factors include the extent of fibrosis and the UIP pattern found on HRCT, and the severity of lung function impairment in terms of diffusing capacity of the lungs for carbon monoxide (DLCO) and forced vital capacity (FVC) [11].

The most common symptoms of RA-ILD include exertional dyspnea and non-productive cough. Bibasilar inspiratory crackles may be commonly revealed on clinical auscultation [15, 16]. In the advanced phase of disease edema, cyanosis and signs of pulmonary hypertension (PH) may also be observed.

PFTs may show a restrictive pattern, with a reduction in FVC—with or without decreased values of DLCO. In a previous study by Gabbay et al. [17], DLCO reduction was reported as a highly sensitive factor for predicting the severity of ILD. Moreover, similarly to IPF, a decrease in FVC of ≥ 10% or a decrease in the DLCO values of ≥ 15% over 6–12 months, has been associated with increased mortality.

#### Radiological features

**Fig. 1:**
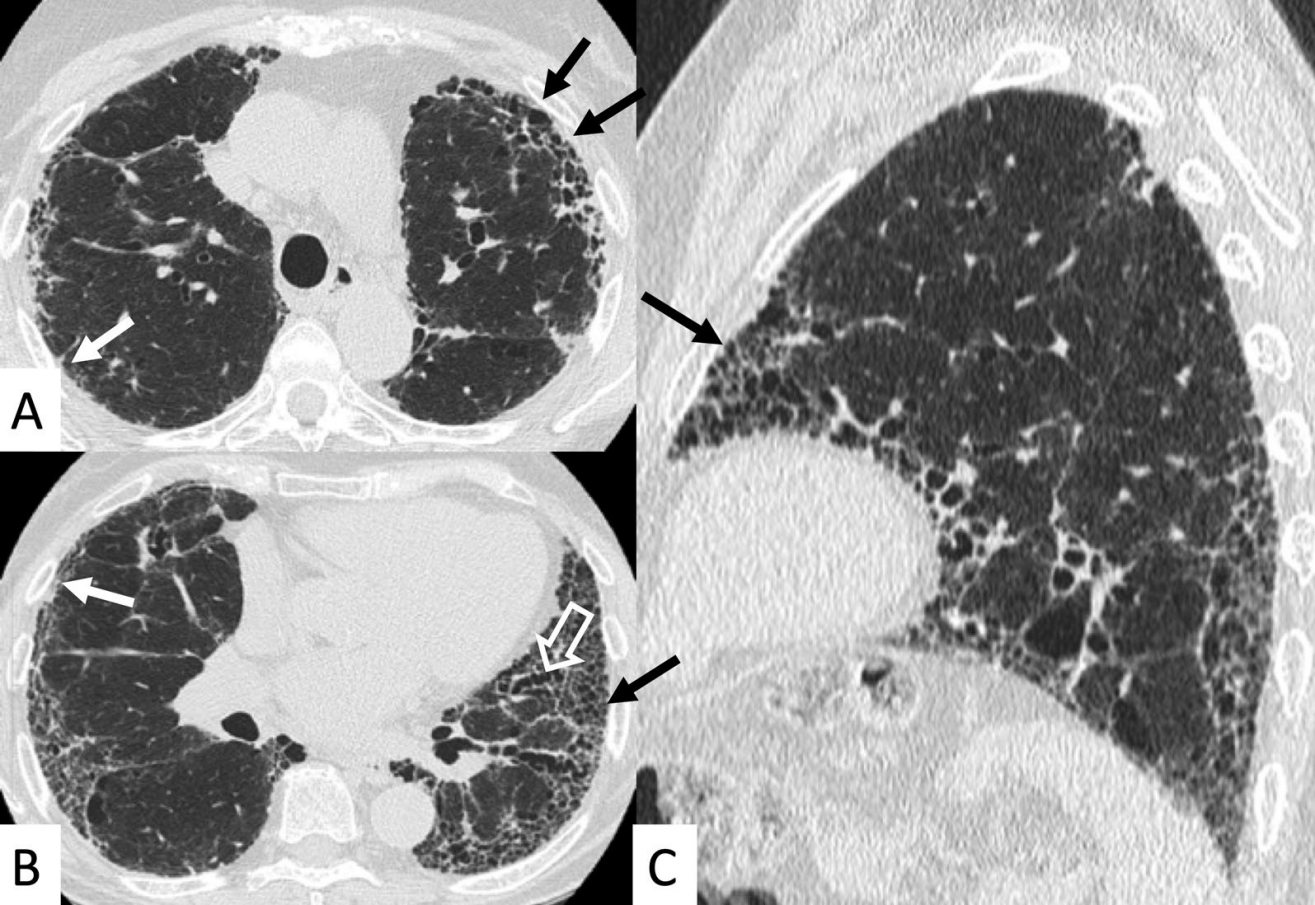
Axial (**A**, **B**) and sagittal (**C**) HRCT images of a UIP pattern in a 72-year-old patient with rheumatoid arthritis. The images show the typical UIP pattern, predominantly located on basal and subpleural regions, and represented by honeycombing areas (black arrows in **A**–**C**), reticulations (white arrows in **A**, **B**) and traction bronchiectasis (white empty arrow in **B**)

UIP represents the most common radiological and pathological pattern encountered in patients with RA (Fig. 1). It has important prognostic implications, being associated with worse survival than fibrotic NSIP appearance; the prognosis, however, is better than patients having UIP pattern due to IPF [11]. Recently published data suggest that a subgroup of RA-ILD patients with UIP may have stable disease for a long term, so that these patients need to be differentiated from those with IPF [7].

**Fig. 2:**
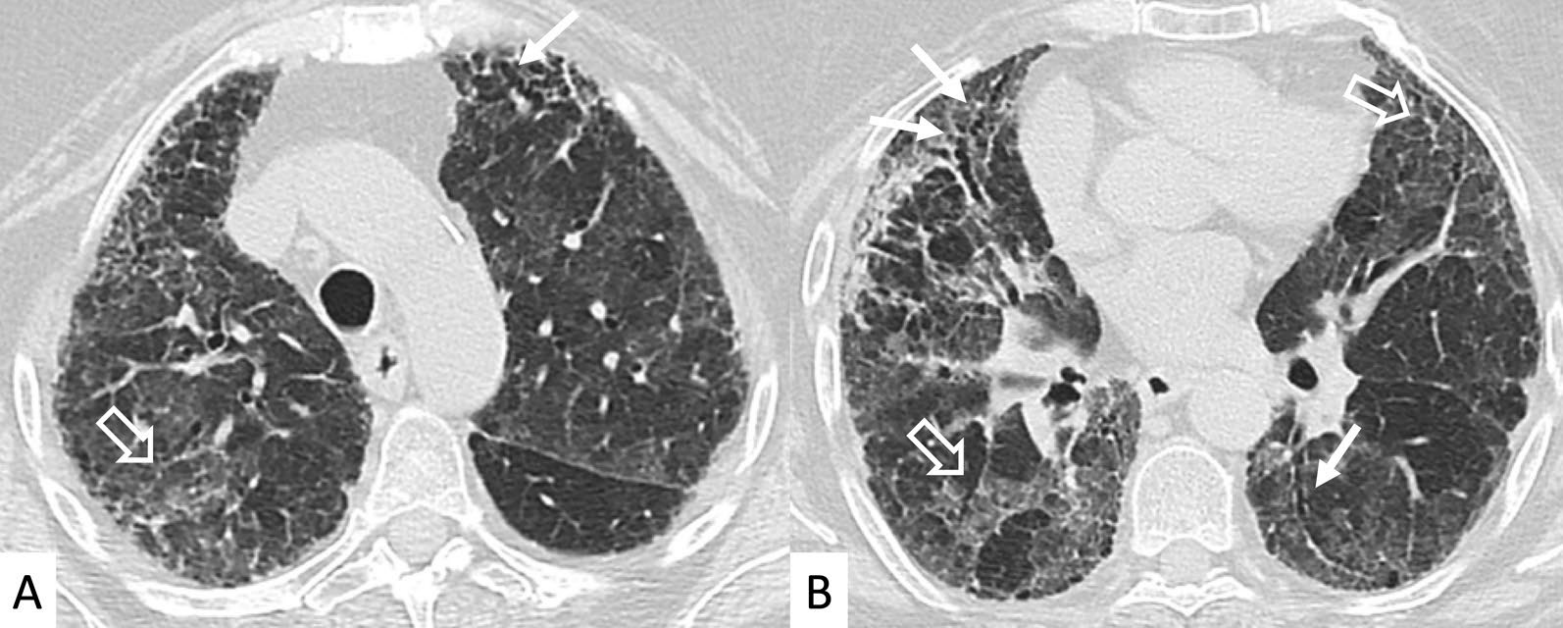
Axial HRCT images of a patient with rheumatoid arthritis. **A**, **B** clearly show ground-glass opacities (empty white arrows) and bronchiectasis (white arrows), resembling an NSIP pattern

**Fig. 3:**
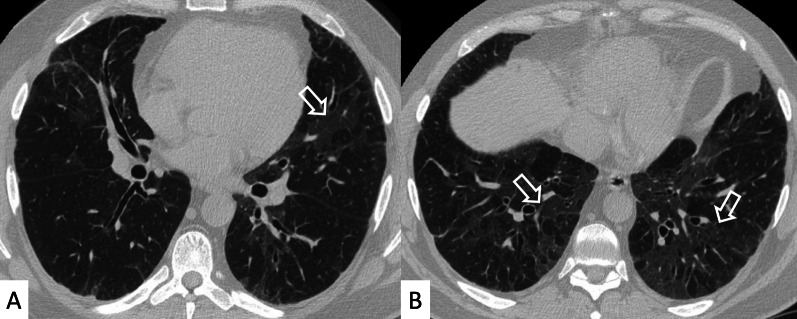
Axial HRCT images of an NSIP pattern in a patient with rheumatoid arthritis. **A**, **B** Ground-glass opacities (empty white arrows) can be appreciated through the pulmonary parenchyma. The heterogeneous distribution of ground glass regions reflects areas of inflammation—alternated to normal lung regions

NSIP, cellular or more commonly fibrotic, can also be observed on HRCT (Figs. 2, 3) [18]—followed by OP with/without interstitial lung fibrosis, lymphocytic interstitial pneumonia (LIP) pattern, and, infrequently, a desquamative interstitial pneumonia (DIP) pattern [18].

The histologic landmarks of RA-associated NSIP or UIP are identical to those observed in idiopathic NSIP and UIP, although interstitial lymphoid aggregates—either within alveolar septal walls or associated with small airways—appear more evident. In contrast, the frequency of fibroblast foci is lesser in RA-UIP than in idiopathic UIP [19].

The radiographic appearance consists of a fine reticular pattern, usually involving the lower lung zones and becomes more diffuse during the progression of disease, when also honeycombing and traction bronchiectasis can be depicted [13].

**Fig. 4:**
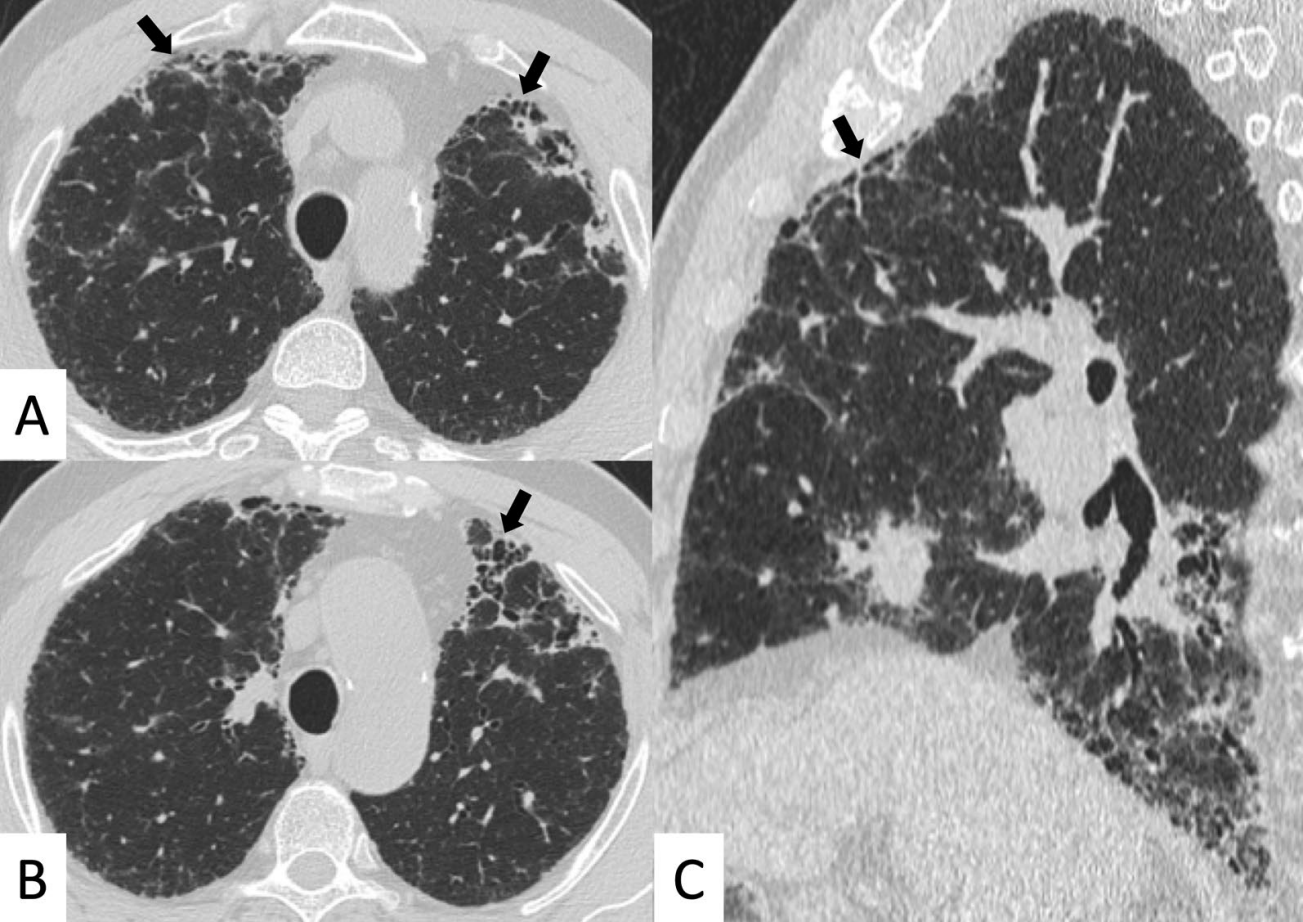
Anterior upper lobe sign in a patient with RA. It consists of a particular fibrosis distribution, most concentrated along the anterior side of the upper lobes; it can be appreciated on axial (black arrows in **A**, **B**) and sagittal MPR image (black arrow in **C**)

**Fig. 5:**
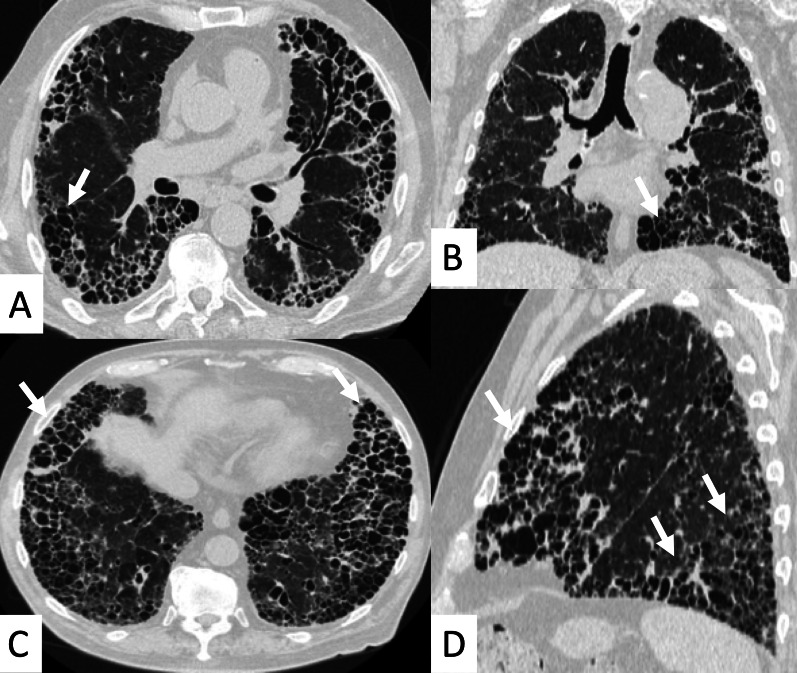
Axial (**A**, **C**), coronal (**B**) and sagittal (**D**) HRCT images demonstrate—in an RA patient—the presence of a florid honeycombing, involving more than 50% of the lung. This finding is typically due to macrocystic spaces (white arrows) and has been defined as “exuberant honeycombing sign”

**Fig. 6:**
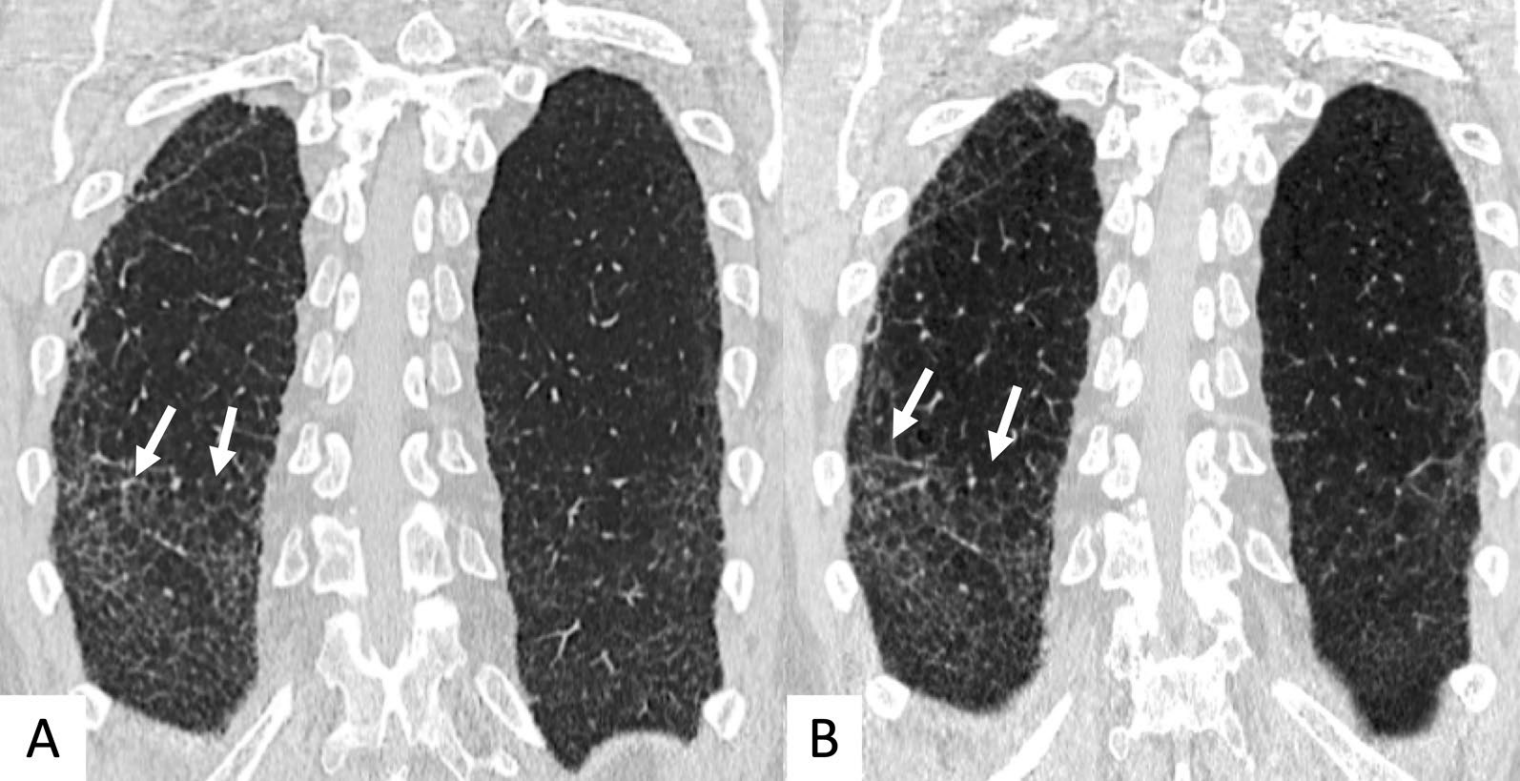
Coronal multiplanar reformatted images. **A**, **B** demonstrate the presence of straight-edge sign in female RA patients showing interstitial lung fibrosis. This sign consists of a more pronounced fibrosis in the lung bases—with sharp demarcation in the cranio-caudal plane (white arrows in **A**, **B**)

However, some typical HRCT findings may be observed in patients with CTDs-UIP: they include the "anterior upper lobe" sign (Fig. 4), the "exuberant honeycombing" sign (Fig. 5), and the “straight-edge” sign (Fig. 6) [20].

The anterior upper lobe sign consists of a particular fibrosis distribution, most concentrated along the anterior side of the upper lobes; in some cases, it is associated with a concomitant lower lobe distribution. The so-called exuberant honeycomb-like cyst formation, represented by the presence of hypertrophic pulmonary honeycomb appearance—due to multiple cysts distributed in several layers—is different from the classic honeycomb, which contains cysts ranging from 3 up to 10 mm [21]. This exuberant appearance is reproduced by larger cysts, and very often is not associated with other fibrotic changes in the remaining lung regions. The straight-edge sign consists of an isolated fibrosis predominantly located in the lung bases—with sharp and clear demarcation in the cranio-caudal plane, easily appreciated on coronal reconstructed images (Fig. 6) [20].

**Fig. 7:**
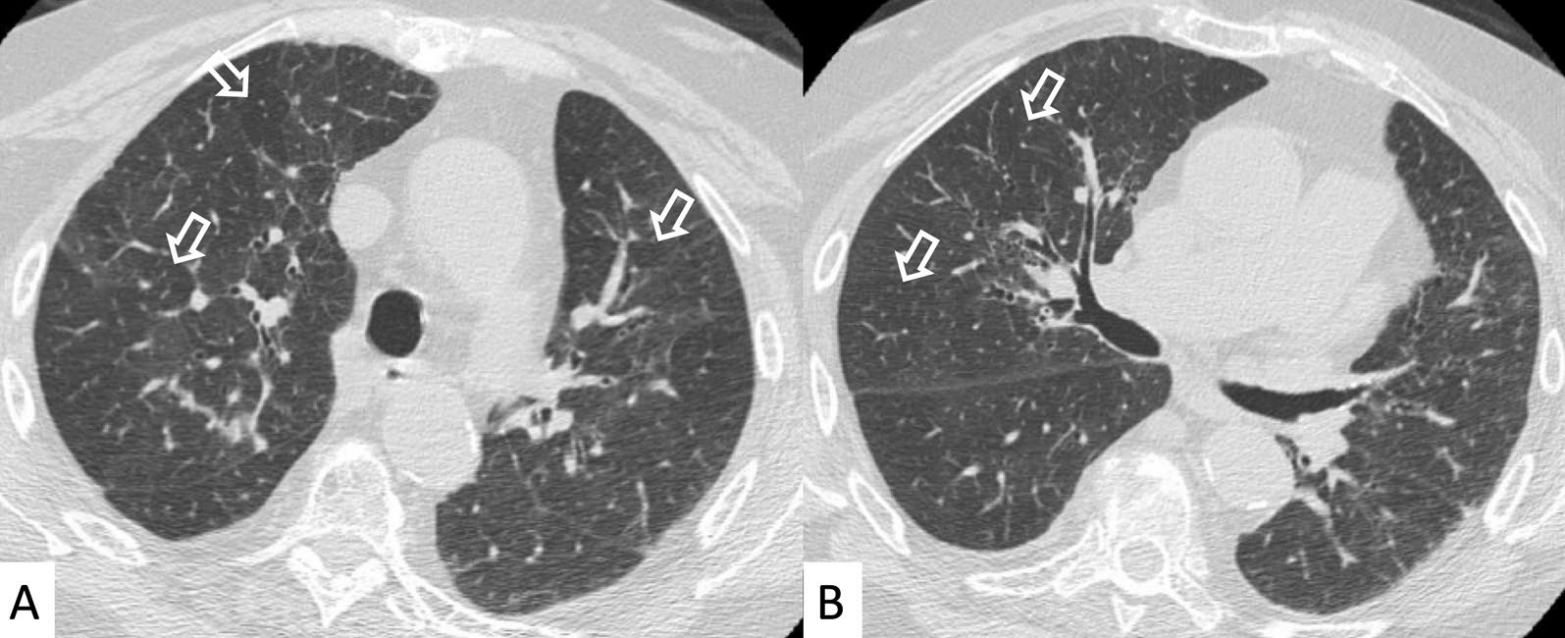
Airway disease in RA patient, clearly depicted in **A**, **B**. Lobular decreased attenuation areas (white empty arrows) are caused by follicular bronchiolitis and obliterative bronchiolitis. This finding represents an early manifestation of disease, due to airway involvement—typically found when no fibrosing alterations are observed

**Fig. 8:**
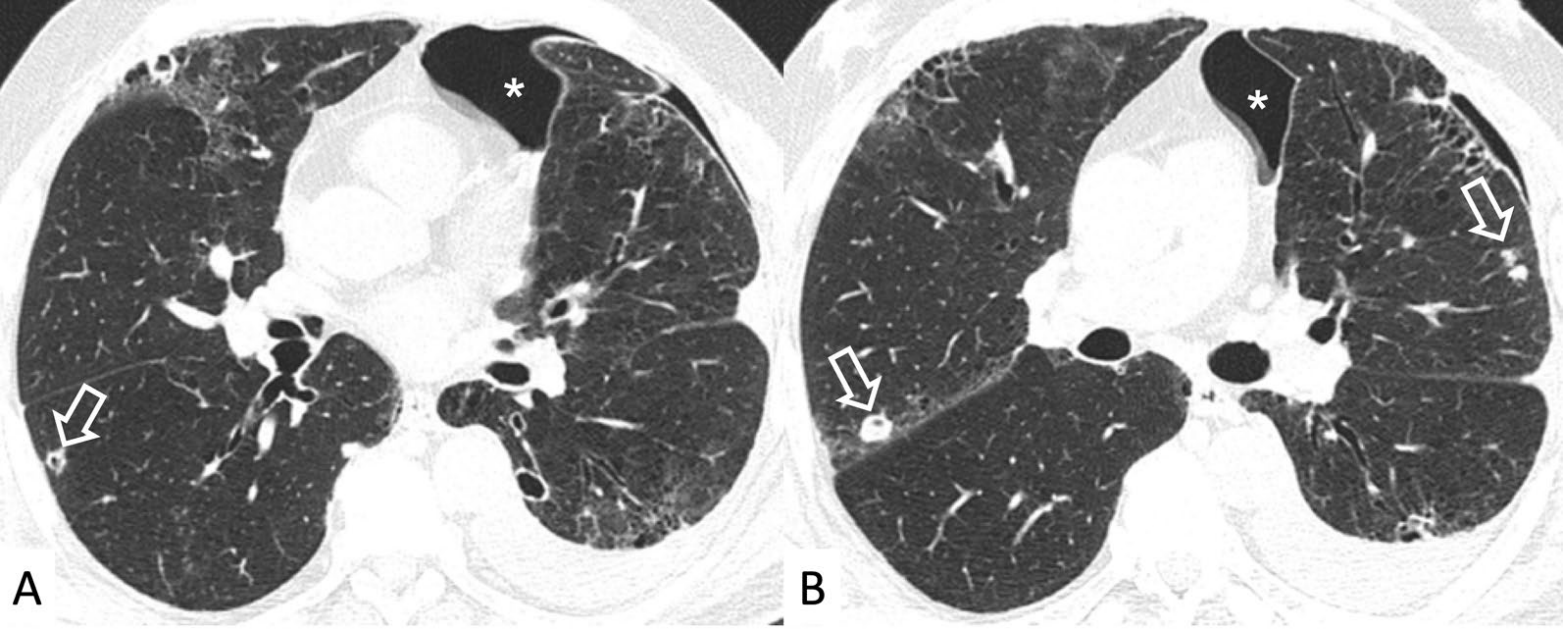
Cavitate nodular lesions (white empty arrows in **A**, **B**) in a male RA patient. Rheumatoid nodules represent a rare manifestation of disease; generally, they have bilateral and subpleural location, with rounded shape. The development of cavitation has been reported in literature related to vasculitis. Left pneumothorax (white asterisk) is also evident along the anterior part of the lung

Another HRCT feature of RA, considered as a secondary finding in a background of NSIP or UIP, is represented by airway disease, such as obliterative and follicular bronchiolitis [22]. This pulmonary alteration seems to be the earliest manifestation of RA in the lung. More in detail, obliterative bronchiolitis is due to the destruction and fibrotic replacement of the bronchioles, which leads to development of air trapping and reproduces a mosaic attenuation (Fig. 7); follicular bronchiolitis is characterized by the occlusion of bronchioles due to the presence of lymphoid aggregates in their walls, with a centrilobular nodules pattern. Rheumatoid nodules are an uncommon manifestation of RA: they are usually round, well-defined, peripherally located, and may cavitate (Fig. 8).

PH commonly occurs in patients with RA, but is usually mild. Other complications of RA include lymphoma, and lung cancer [22].

### Systemic sclerosis (SSc)

SSc—also known as scleroderma—is an autoimmune CTD characterized by fibroblast dysfunction and deposition of excessive extracellular matrix, leading to microvascular damage and progressive fibrosis of the skin and internal organs [23]. It is an uncommon disease, with an incidence of approximately 10 cases per million per year; it occurs more frequently in women between 30 and 70 years [13].

According to the extent of skin involvement, SSc has been divided into two forms [24, 25]: (1) *limited SSc* (skin thickening proximal to elbows and knees, but not involving the trunk), and (2) *diffuse SSc* (skin thickening of trunk, shoulder, pelvis and face), having also different involvement of internal organs and autoantibody subsets.

In addition to the skin thickening, disease is characterized by a wide spectrum of manifestations—which includes telangiectasias, fingertip ulcers, gastrointestinal diseases, myocardial diseases, and pulmonary involvement [26]. A joint ACR-EULAR (American College of Rheumatology-European League against Rheumatism) committee has developed new classification criteria for SSc diagnosis, based on the application of a point-system score ranging from 9 up to 19. In this classification, main clinical feature is considered the skin thickening of the fingers of both hands that extends proximal to the metacarpophalangeal joints. Other domains include skin thickening of the fingers, fingertip lesions, telangiectasia, abnormal nailfold capillaries, PH and/or ILD, Raynaud Phenomenon, Scleroderma related antibodies—mainly represented by anti-centromere antibodies (ACA) and anti-Scl-70 antibodies [27].

A score of 9 provides a definite diagnosis, and can be obtained with the presence of the main domain only (skin thickening extending proximal to metacarpophalangeal joints), or by the sum of the other clinical and serological domains [27].

#### Lung involvement

Most common pulmonary manifestations are represented by ILDs—often observed in the diffuse form of SSc [28], and pulmonary arterial hypertension—which may be found more commonly in the limited form of disease, as an isolated finding or in association with lung fibrosis [14, 22]. The prevalence of these manifestations ranges from over 22% (especially for PH) to up to 80% (ILD) [29].

Pulmonary involvement is frequently observed and is associated with high morbidity and mortality [30]. Among patients with collagen vascular disease, those with SSc show the highest mortality, due to the development of pulmonary hypertension—which has a prevalence ranging from 10% up to 16% [31]. ILD development, which shows the highest prevalence in SSc than in other CTDs, represents the main cause of mortality in SSc, being responsible for up to 40% of the causes of death [32, 33]. In other CTDs, the main cause of death is related to cardiovascular disease (RA and Sjogren’s syndrome) [34, 35], infection or malignancy (IIMs) [36].

In contrast with other CTDs, the pulmonary involvement in SSc tends to be limited to the interstitium, with or without vascular involvement; pleural and airways diseases are rarely observed.

As previously reported, a higher risk of ILD can be related to the diffuse form of SSc, and is associated with the presence of antibody profiles. More in detail, the presence of anticentromere antibodies often suggests limited skin involvement and absence of lung involvement, whereas anti-Scl-70 antibodies increase the risk for diffuse skin involvement and scleroderma lung disease [37].

The Th/To and PM-Scl antibodies have been related to the presence of limited skin disease [38]; they represent a marker for the development of hypertension [37]. Additional risk factors for ILD include esophageal dysmotility and gastroesophageal reflux disease. Specific patient features (old age, male gender, lower baseline FVC, and low baseline DLCO), imaging findings (extent of ILD on HRCT) and time of disease (shorter duration of scleroderma before the onset of respiratory symptoms)—seem to be predictive factors of progression and mortality in CTD-SSc [22, 39].

From the clinical point of view, symptoms of SSc-ILD include exertional dyspnea, non-productive cough and fatigue. Generally, the clinical examination reveals “velcro” crackles—which cannot be attributed to other causes. PFTs show a restrictive ventilatory impairment, with a low DLCO value [40].

#### Radiological features

**Fig. 9:**
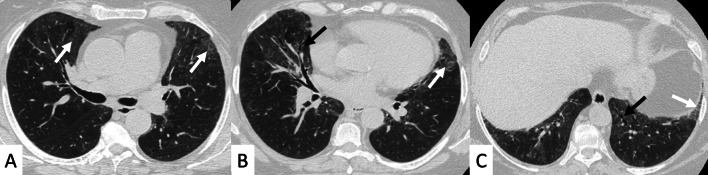
Typical NSIP pattern in an SSc patient. **A** Ground-glass opacities and fine reticulations (white arrows), predominantly located in peripheral regions; in **B**, **C** traction bronchiectasis are also depicted (black arrows)

**Fig. 10:**
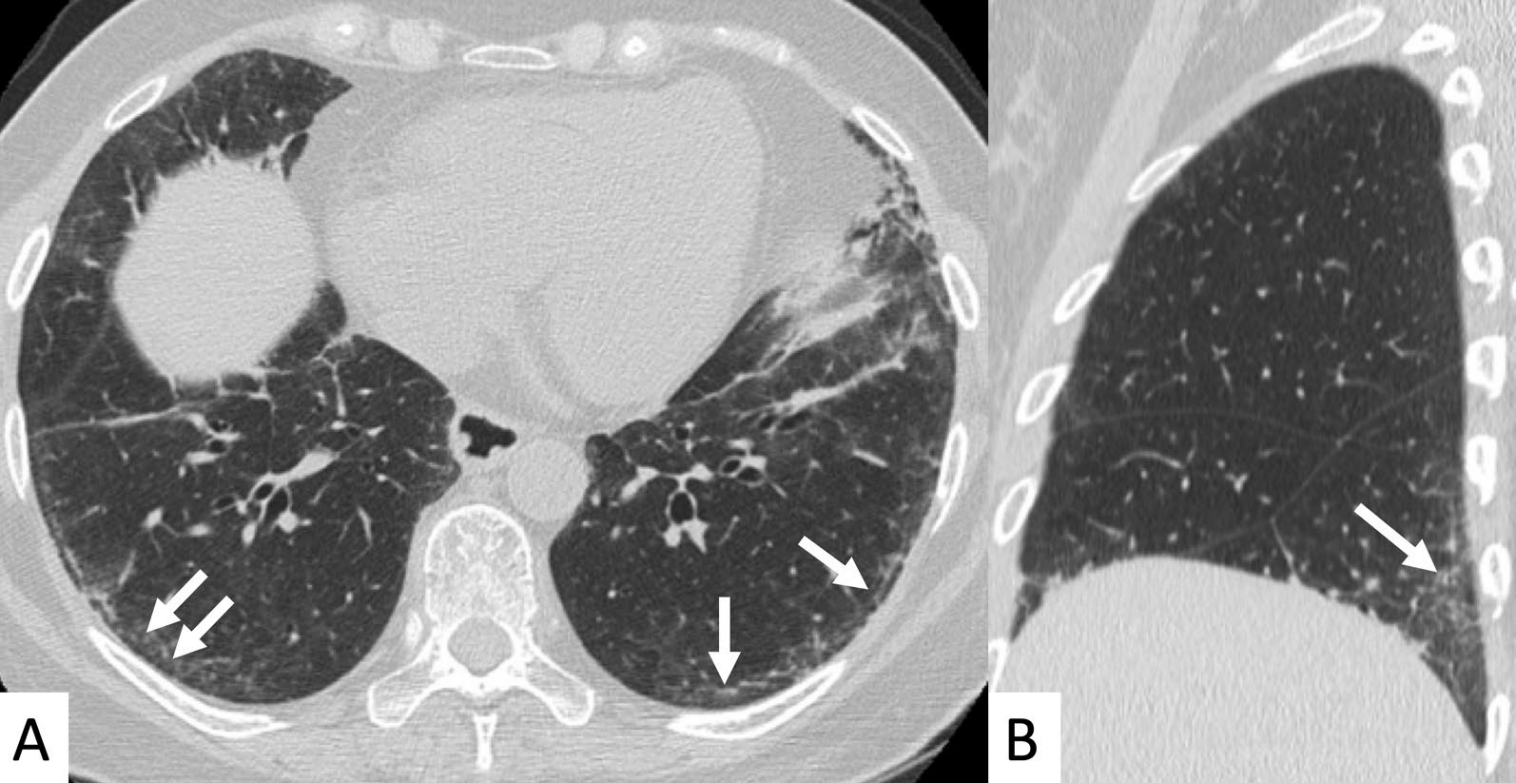
NSIP pattern with subpleural sparing in a female SSc patient. Axial (**A**) and sagittal (**B**) HRCT images demonstrate ground-glass opacities predominantly located in the basal pulmonary regions, with peripheral and subpleural distribution (white arrows). Pulmonary alterations may spare subpleural regions, as well demonstrated in **A**—namely in the left lower lobe

**Fig. 11:**
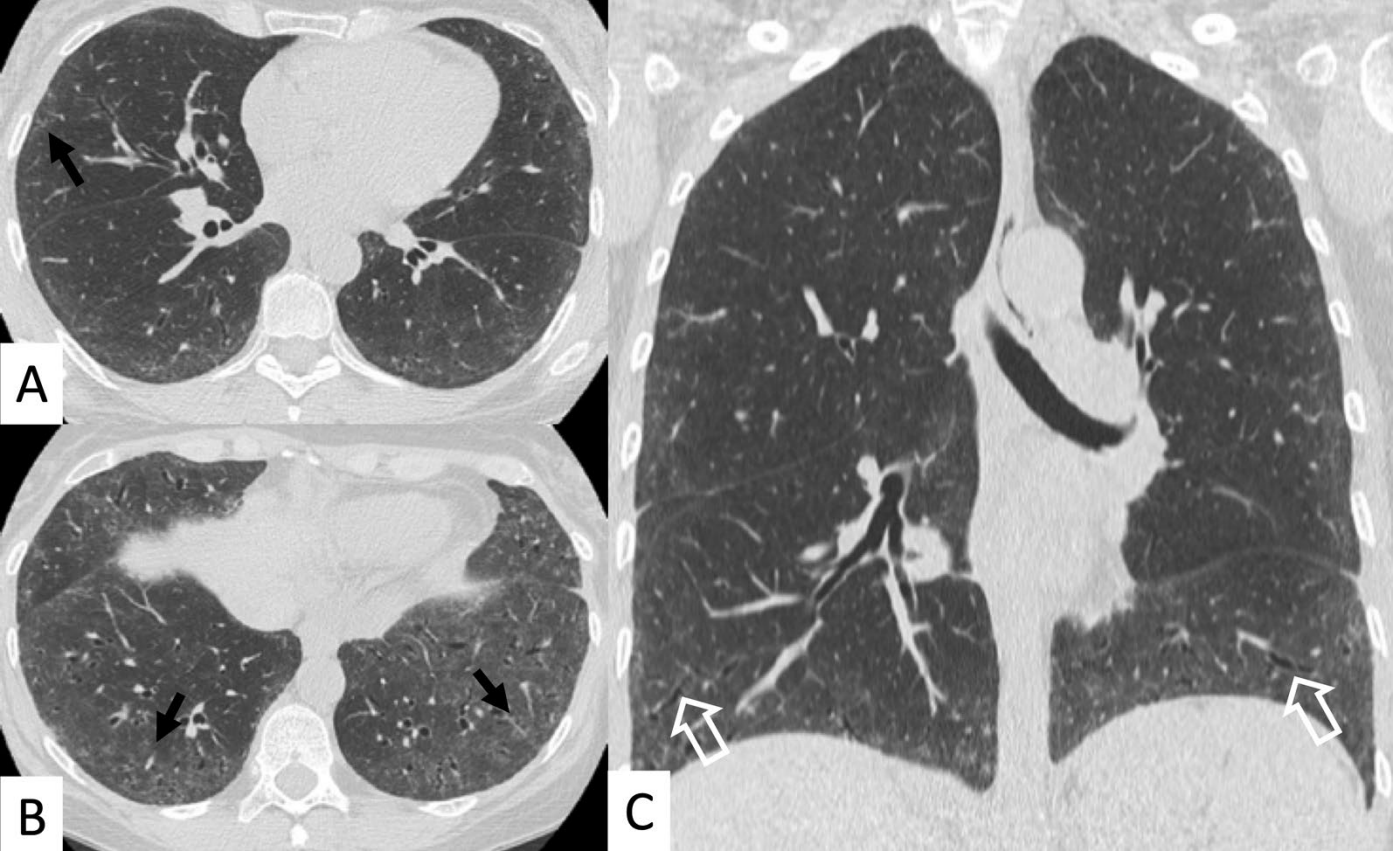
Axial (**A**, **B**) and coronal (**C**) HRCT images of an SSc patient demonstrate NSIP pattern with ground-glass opacities (black arrows), well depicted in the subpleural and basal regions of the lung; fine reticulations may be also observed. Coronal MPR image is useful to better delineate the presence of small bronchiectasis, which may be seen in the peripheral basal regions (white empty arrows)

NSIP is the most common pattern of interstitial fibrosis found in SSc (Fig. 9), confirmed by several recent studies, including the largest one published by Bouros et al. – which recruited a total of 80 SSc-ILD patients [10, 41]. This uniform and diffuse fibrosis frequently involves lower lobes with a predominant posterior and peripheral distribution [31], often sparing the immediate subpleural area (Fig. 10). More in detail, in cases of NSIP pattern we observe a greater proportion of ground-glass opacities (GGOs) and a lower degree of coarse reticulation [42] (Fig. 11); fine reticulations could be appreciated, usually associated with traction bronchiectasis and bronchioloectasis (Fig. 11). Honeycombing, when present, is usually mild—even if when present it should be considered a sign of disease progression, with the replacement of ground-glass areas with honeycomb, and development of “end-stage lung disease” [19, 43]; the “straight-edge” sign is more common in SSc than RA. According to a study by Kim et al., the progression rate of honeycombing in patients with SSc is 0.07% of lung volume per month [44]. Moreover, a recent study found a correlation between PFTs and HRCT features (honeycombing and GGO) during follow-up examinations: in particular, the increase of honeycombing extension seems to be significantly correlated with DLCO decrease [31, 45].

**Fig. 12:**
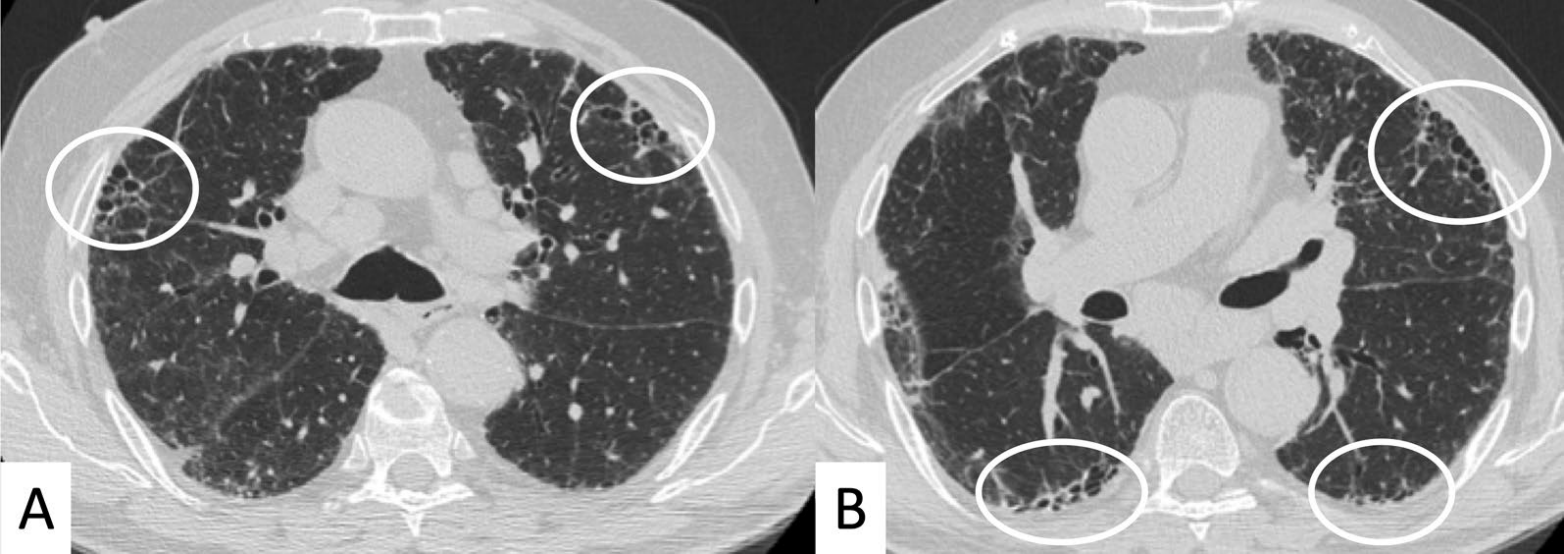
The Four Corner Sign. This sign is defined by the presence of pulmonary fibrosis in the “4 corners regions of the thorax”—represented by anterolateral regions of mid-upper lobes and the posterosuperior regions of lower lobes. In this SSc patient, honeycomb areas are clearly recognizable in the upper lobes (white circles in **A**, **B**) and in the upper parts of lower lobes (white circles in **B**)

Another useful CT finding is represented by the “Four Corners” sign (Fig. 12), defined by the presence of reticular opacities, ground-glass attenuations, and/or honeycombing distributed in the “4 corners regions of the thorax”: the anterolateral regions of mid-upper lobes (from the top of the aortic arch to the carina) and the posterosuperior regions of lower lobes (carina through the inferior pulmonary veins) [29]. In the retrospective HRCT analysis of 116 IPF and 115 SSc-ILD cases published by Walkoff et al. [29], a pattern of focal or disproportionate inflammation and/or fibrosis involving the bilateral anterolateral upper lobes and posterosuperior lower lobes (the four corners sign), was more commonly reported in SSc-ILD patients, with a specificity of 100% and a sensitivity of 16.4% [29].

The four corners sign differs from the propeller sign: The latter is represented by a continuous gradient of peripheral fibrosis (honeycomb and/or reticulation) in UIP pattern—with a “propeller blade” distribution on axial images moving posterior to anterior from lung bases toward the apex [46]. Sagittal views may help radiologists in confirming or excluding the focal distribution of these signs, even if a clear differentiation between the four corners sign, anterior upper lobe sign and propeller sign may not be easily achieved. In addition, the low sensitivity reported in the study by Walkoff et al. [29], reduces the real utility of this sign in the differentiation between IPF and secondary fibrotic patterns.

A typical UIP pattern – with honeycombing, lung volume loss, and temporal and spatial heterogeneity of the lesion—may sometimes be seen, being depicted in up to 33% of cases [42]; however, as reported by Bouros et al., it has not been associated with a worse prognosis compared to NSIP [10, 41].

Interestingly, as reported by De Lauretis et al., “non-UIP/non-NSIP patterns”, such as follicular bronchiolitis, diffuse alveolar damage (DAD) and OP patterns, “are quite rare in the context of SSc” [10]; they may be occasionally found on HRCT images.

The PH prevalence is variable, depending on the method of detection; values ranging from 13% up to 35% have been reported using transthoracic Doppler echocardiography [42], and ranging from 8% up to 12% using right heart catheterization [43]. The histological hallmark of this vascular change is represented by concentric intimal thickening due to fibromyxoid tissue and mild medial hypertrophy—leading to thickened and stenotic pulmonary arterioles. The severity of vascular changes is not always proportionally associated with the degree of interstitial fibrosis [19].

**Fig. 13:**
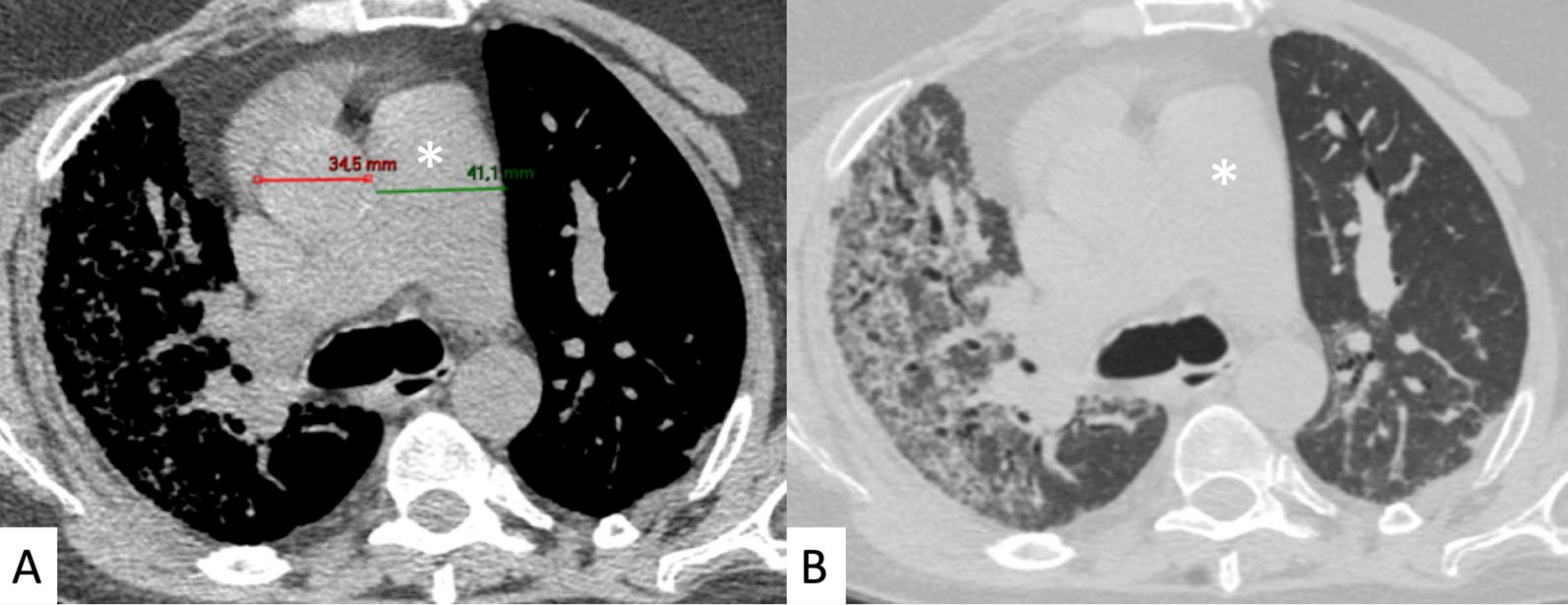
A patient with pulmonary hypertension (PH) assessed by chest CT. **A** Mediastinal window preset of visualization and (**B**) lung windowing preset of visualization clearly demonstrate the enlargement of the pulmonary trunk (white asterisk); commonly, the main pulmonary artery/ascending aortic/ratio is calculated. Generally, normal values are less than 1.0, whereas in cases of superior values, PH may be suspected

**Fig. 14:**
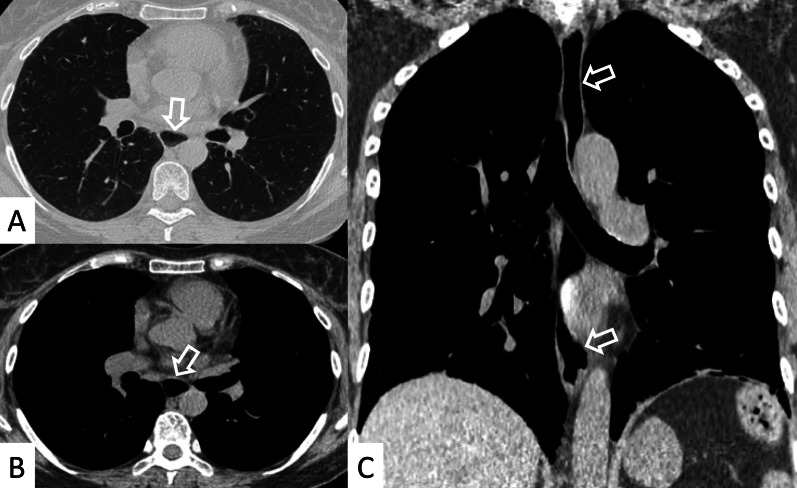
Axial (**A**, **B**) and coronal (**C**) images of an SSc patient. Dilatation of the esophagus is clearly recognizable, being depicted (white empty arrows) in the middle and distal parts of the mediastinum

Radiological features on chest radiograph or HRCT include enlargement of main and proximal pulmonary arteries (Fig. 13), although a normal-sized pulmonary artery does not exclude the diagnosis. The presence of pericardial thickening or effusion is also a strong predictor of echocardiographic pulmonary hypertension. In 80% of scleroderma cases, dilatation of distal two-thirds of the esophagus is observed [47] (Fig. 14).

Most of the patients have relatively limited disease, so they remain stable without treatment. To identify “a progressive pattern”, Goh et al. introduced a staging system based on the assessment of disease severity [48]. A semiquantitative HRCT analysis allows classifying patients in extensive or limited disease—depending on whether CT extent is deemed to be greater or less than 20%, respectively. Only if this division is uncertain, PFTs play a role in assessing the degree of disease severity, according to whether FVC is lower or higher than 70% of predicted [48].

### Polymyositis (PM) and dermatomyositis (DM)

PM and DM are autoimmune inflammatory diseases, characterized by proximal muscle weakness, systemic involvement and specific cutaneous manifestations [49]; their incidence ranges from 1.2 to 66 new cases per 1,000,000 person-years, with female predilection [50].

According to a variable degree of muscle inflammation and systemic involvement, the appropriate term to define these conditions is idiopathic inflammatory myopathies (IIMs)—a group of CTDs which may also include the antisynthetase syndrome (ASSD) [51].

Diagnostic criteria for IIMs, firstly proposed by Bohan and Peter, include: (1) symmetric proximal muscle weakness, (2) serum elevation of the skeletal muscle enzymes such as creatine kinase (CK), (3) characteristic electromyography alterations, (4) muscle biopsy with evidence of necrosis and (5) typical skin manifestations for DM, such as heliotrope rash and Gottron papule [52, 53].

#### Lung involvement

Pulmonary involvement, and namely ILDs, are the most common non-musculoskeletal manifestations of IIMs [51]. Lung involvement occurs in 30–66% of patients with myositis, and represents the major cause of morbidity and mortality [10]; as reported by Vacchi et al., “ILD results often in decreased quality of life and increased utilization of healthcare services” [54]. In these CTDs, it may manifest in one or more of three forms:

1. Hypoventilation and respiratory failure due to the involvement of the respiratory muscles;
2. Interstitial pneumonitis, usually showing NSIP pattern or OP pattern;
3. Aspiration pneumonia—secondary to pharyngeal muscle weakness (the most common pulmonary complication) [13, 55].

Other pulmonary complications, such as hypertension and pneumomediastinum, have been less frequently described in patients with myositis [56].

The prevalence of IIM-ILDs ranges from 20 to 78%—depending on methods of ascertainment and patient selection [14]. The ILD development usually may precede the onset of clinical myositis, and is associated with increased mortality [56]. Therefore, risk factors for ILD development should be early identified: older age, presence of arthritis/arthralgia, “mechanic’s hands”, ulceration and elevated levels of erythrocyte sedimentation rate and C-reactive protein, are all associated with increased risk of ILD [57, 58]. Furthermore, autoantibodies against aminoacyl-tRNA synthetases (anti-synthetases) have been strongly linked to ILD; namely, anti-histidyl-tRNA synthetase (Anti-Jo1) antibodies is the most common anti-synthetases, depicted in 25–40% of patients with PM/DM, and in 30–75% of the ILD subgroup. Also, serum levels of KL-6, a marker of epithelial cell damage/regeneration used to monitor disease activity, have also been considered a predictive factor for ILD development [56]. Respiratory symptoms may develop after, concomitantly, or before the onset of muscle and skin manifestations, occurring at any time during the course of the diseases [59].

The overall clinical course in patients with IIMs-ILD can be quite variable, ranging from subclinical to rapidly progressive forms [14]; progression is more likely observed in patients with lower FVC and symptomatic disease [56].

In symptomatic ILDs, two main clinical scenarios may be encountered: (a) pulmonary infiltrates with rapidly progressive dyspnea and frequent evolution into respiratory failure; (b) insidious onset of dyspnea with radiological abnormalities that represent the most common pattern.

When ILD is the only pulmonary manifestation, PFTs may show a moderate restrictive pattern, with decreased values of DLCO and FVC. The concomitant presence of respiratory muscle inflammation, especially diaphragmatic weakness, can also result in changes in PFTs [56]; however, when the muscle strength is less than 50%, a reduced lung volume may be found [10].

#### Radiological features

**Fig. 15:**
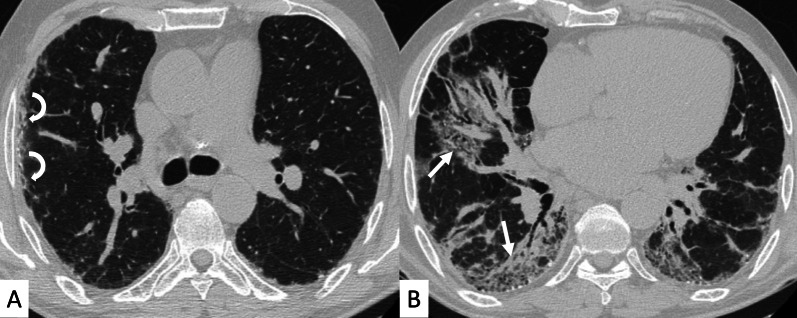
NSIP pattern in a patient with PM. Subpleural reticulations are located in the subpleural regions, namely located in the right lung (white curved arrows in **A**. Ground-glass opacifications and reticulations are demonstrated through the lung, predominantly located in the basal right lung and in the middle lobe (white arrows in **B**), with small bronchiectasis included

**Fig. 16:**
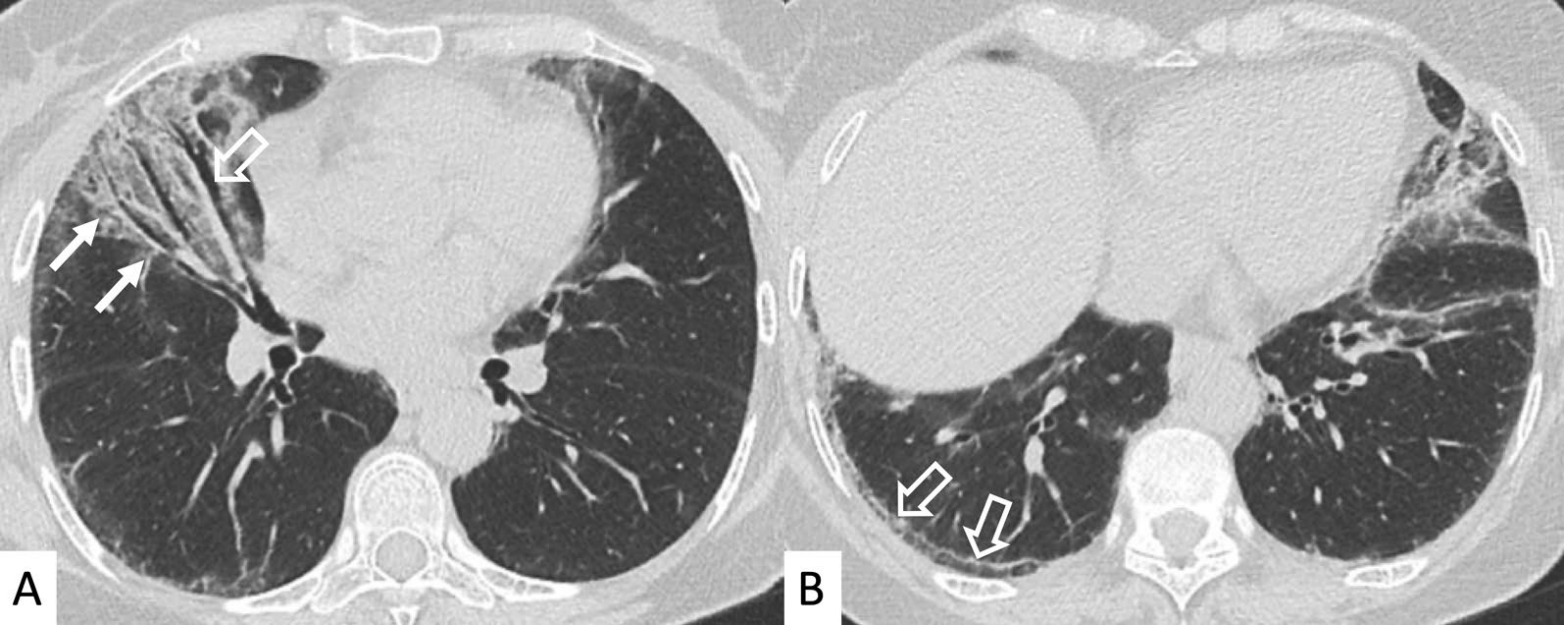
NSIP pattern in a patient with PM. HRCT features include reticulations superimposed on ground-glass opacifications, as clearly depicted in the middle lobe (white arrows); traction bronchiectasis (empty white arrow) can also be appreciated in the same area. Subpleural reticulations are also evident in **B**, in the basal part of the right lung (empty white arrows)

**Fig. 17:**
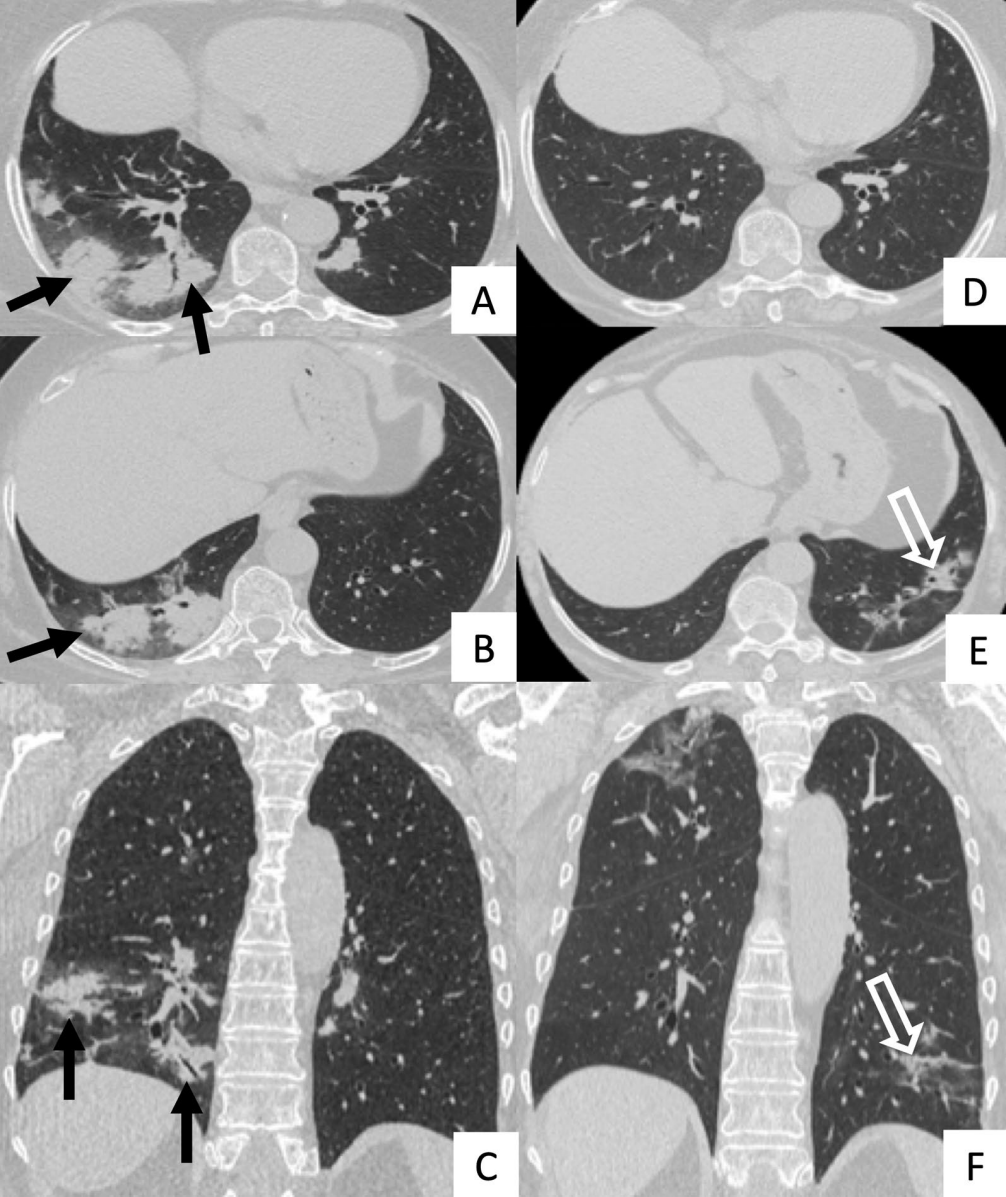
Axial (**A**, **B**) and coronal (**C**) HRCT of a PM-DM patient, showing OP pattern. **A**–**C** Peripheral and bronchocentric consolidations (black arrows); the “air bronchogram sign” is clearly recognizable. Follow-up HRCT images (**D**–**F**) demonstrate the typical evolution of organizing form, with tendency to migration and location changing (empty white arrows)

Most frequent ILD patterns—associated with PM or DM are represented by NSIP (Figs. 15, 16), OP (Fig. 17) and DAD [55]. However, patients with antiJo-1 antibodies may exhibit UIP pattern: in these cases, a differential diagnosis with IPF can be challenging—namely if myositis is clinically absent. To provide a correct diagnosis, an accurate serological assessment is required [51]. Histologic appearance is helpful in determining the prognosis: patients with DAD or UIP have a poor prognosis, with only a 33% survival rate at 5 years; patients with OP and/or NSIP patterns have, respectively, excellent and good prognosis [13].

At chest radiographs, parenchymal abnormalities include the presence of a reticular pattern, with basal and symmetric distributions [60]; bilateral areas of consolidations may develop in some patients, suggesting superimposed DAD and/or OP patterns [13].

**Fig. 18:**
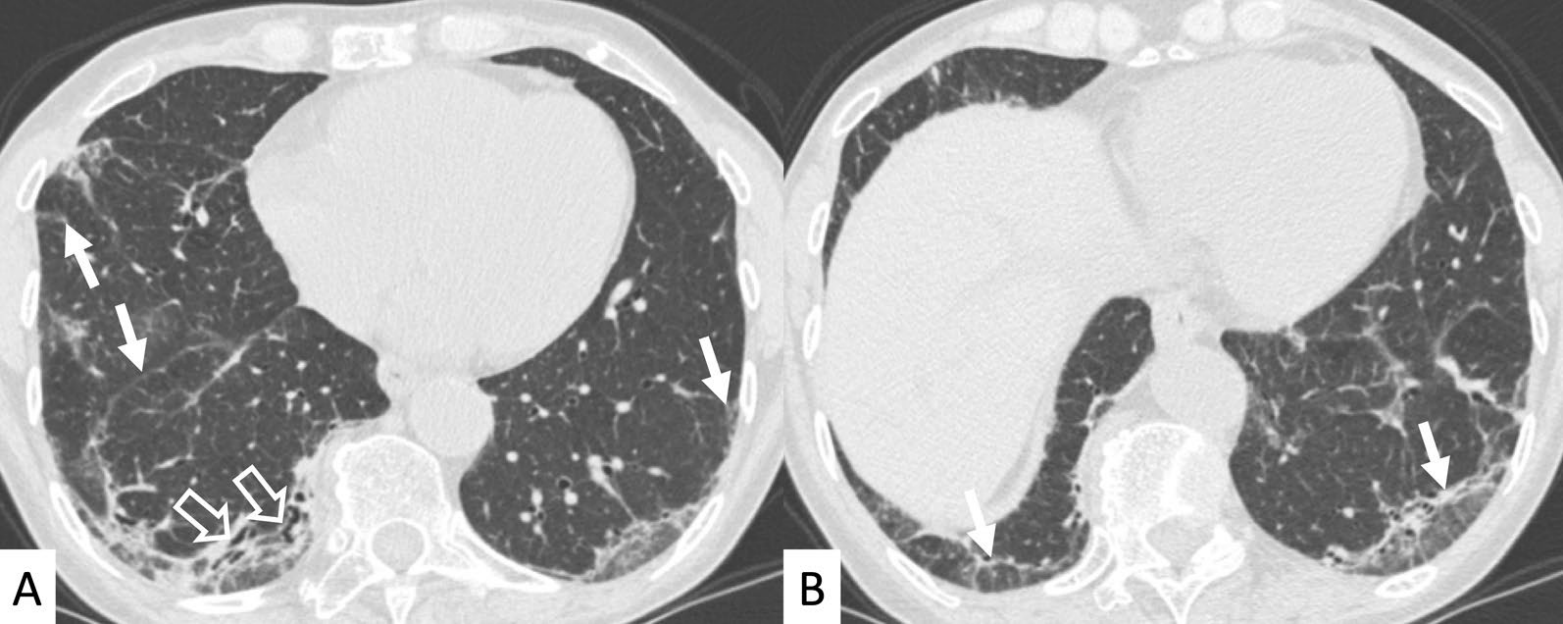
OP and NSIP pattern in a clinical case of antisynthetase syndrome. Bronchiectasis are clearly seen in the dorsal region of the right lung (white empty arrows in **A**); small linear opacities and lines (white arrows) are also appreciable in **A**. Subtle consolidations resembling a perilobular distribution are well depicted in **B** (white arrows)

ILDs associated with PM/DM or ASSD syndrome are often characterized by a typical HRCT appearance, which consists of confluent GGO areas and consolidations located in the lower lobes, and superimposed on a reticular pattern with traction bronchiectasis; honeycombing is a rare manifestation, being identified in up to 16% of patients who have abnormal chest radiographic findings or pulmonary function [13]. This appearance reflects the histologic combination of OP and fibrotic NSIP [61] (Fig. 18).

OP pattern can also be revealed as an isolated manifestation: it is defined by the presence of focal granulation tissue in the alveoli and their ducts, which progressively leads to the obstruction of the smaller airways. On HRCT images, it is characterized by patchy areas of ground glass opacities or consolidations, generally showing more alveolar and less bronchocentric distribution—in comparison to OP not associated with IIMs [62].

**Fig. 19:**
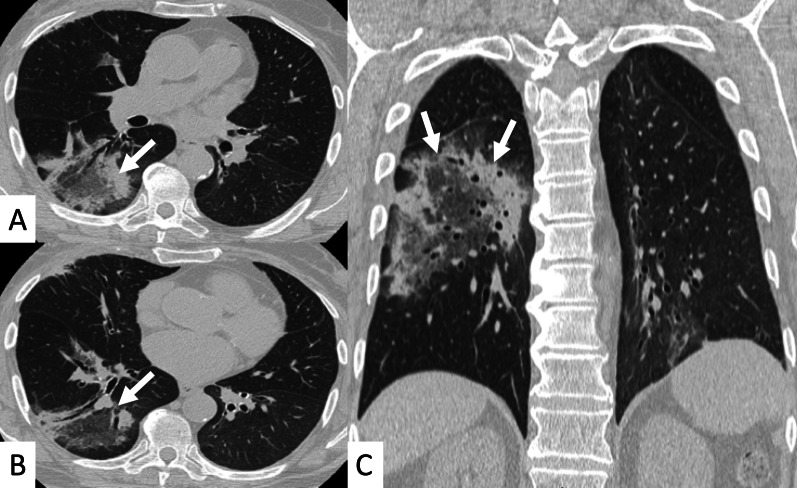
Reverse halo sign or “atoll sign” in a patient with OP. HCRT images show circular consolidations surrounding normal parenchyma (white arrows in **A**–**C**)

In some cases, the "reverse halo" sign is reproduced, with central ground-glass opacities surrounded by parenchymal consolidations (Fig. 19) [22].

**Fig. 20:**
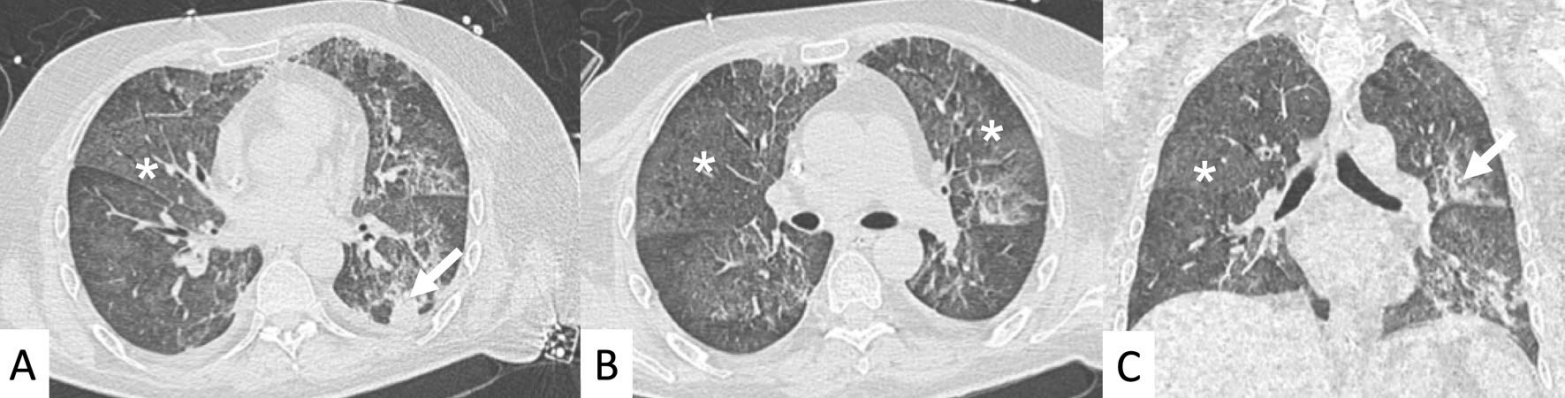
A 62-year-old man with acute respiratory failure. He referred Gottron papules and acral cutaneous ulcerations one month previously; laboratory exams revealed increased Creatine-Phosphokinase levels (800 u/l) and presence of Anti-melanoma differentiation-associated gene 5 (MDA5) antibodies. Diffuse ground-glass opacifications (white asterisks in **A**–**C**) and small consolidations (white arrows in **A**, **C**) are depicted, suggesting HRCT features of diffuse alveolar damage

DAD is characterized on HRCT by diffuse ground-glass opacities and extensive consolidations (Fig. 20); it is associated with a very poor prognosis [10]. Other rare manifestations of ILD associated with PM-DM include pleuritis, airway involvement, and vasculitis [19].

Lung biopsy is not usually performed in patients with PM/DM-ILD, in view of its limited usefulness in the diagnosis and management of the disease [56]. Bronchoscopy with bronchoalveolar lavage (BAL) can be helpful in patients undergoing immunosuppressive treatment in order to exclude infection [10].

### Systemic lupus erythematosus (SLE)

SLE is a chronic autoimmune multisystem disorder of unknown etiology that may affect almost any organ—joints, skin, kidneys, serosa, vessels and the central nervous system; it shows an incidence of 2–3 cases per year per 100,000 persons [63, 64]. Most commonly depicted in young women [13], SLE is characterized by the production of antinuclear antibodies (ANA). Other common serological abnormalities include anti-dsDNA antibodies (76%), hypocomplementemia (71%), and anti-Ro and/or anti-SS-A antibodies (35%) [65].

This disease may have a wide spectrum of clinical manifestations: rheumatological, dermatological, and renal abnormalities are commonly observed as well as pulmonary involvement. The latter may occur with a wide range, being found in 20–90% of patients with SLE [66].

#### Lung involvement

All pulmonary compartments may be affected: pleura (pleural effusion), lung parenchyma (acute pneumonitis, respiratory infections and ILD), respiratory muscles and pulmonary vessel (acute pulmonary hemorrhage). While acute pulmonary lesions (pulmonary hemorrhage and acute lupus pneumonitis) are usually associated with high levels of systemic lupus activity, chronic pulmonary forms of disease—such as interstitial pneumonitis and fibrosis—can progress independently of other organ involvement [65]. Even if pulmonary disease is not included among the diagnostic criteria, lung involvement, namely in the chronic form, has an important negative effect on prognosis. Furthermore, an increased risk of respiratory infections has been related to some SLE therapies [66].

In contrast with the high prevalence of pleuritis and acute pulmonary manifestations (50–60%), fibrotic ILD is less commonly found in SLE than in the other collagen tissue diseases, occurring only in a range of 1–15% of patients [22]. It is considered an unusual finding, and also clinical progression is usually slow, remaining often a stable disease [65]. Risk factors for ILDs include: older age at the initial presentation (> 50 years old), longstanding disease (> 10 years), and the presence of overlapping clinical features with scleroderma—such as Raynaud’s phenomenon, sclerodactyly, abnormal nailfold capillaries and anti-ribonucleoprotein (RNP) antibodies [66]. Hypocomplementemia, high levels of C-reactive protein, and the presence of cryoglobulins or lupus erythematosus cells in the serum have been also associated with increased risk of ILD development. Finally, the interstitial disease may represent a sequel of acute lupus pneumonitis/DAD pattern [10].

A minority of patients with SLE (3–8%) have a clinically significant ILD, with symptoms such as dyspnea and cough that are similar in most types of ILD [22]. In the absence of respiratory symptoms, the diagnosis is usually incidental, achieved through the recognition of radiological findings on chest imaging examination, or through the identification of abnormal lung function tests [66]. PFTs may show a restrictive pattern of disease and, most commonly, a DLCO reduction is observed [65].

#### Radiological features

Pleural involvement could be observed with radiological signs of pleuritis and/or effusion; parenchymal manifestations may be radiologically revealed as pulmonary infections, chronic interstitial lung disease, acute lupus pneumonitis (ALP), and diffuse alveolar hemorrhage (DAH) [67]. In a previous study by Kinder et al., the etiology of pulmonary infections has been related to bacterial pathogens in 75% of cases, mycobacteria in 12%, fungi in 7%, and virus in 5% [67, 68].

Considering the chronic interstitial disease—as with other CTDs—NSIP represents the most frequent radiological and pathological pattern encountered, followed by LIP and OP; the UIP pattern is very uncommonly reported [51]. Bronchiolitis obliterans has been also reported as the initial manifestation of SLE [69].

Some studies have described the presence of mononuclear or lymphocytic interstitial and peribronchiolar infiltrates, which are associated—with variable intensity—in a cellular NSIP pattern having lesser degrees of fibrosis [19].

Radiographic evidence of interstitial fibrosis—characterized by a reticular pattern, predominantly in the lower lobes—is seen in only about 3% of LES patients. Instead, HRCT interstitial abnormalities are seen in 30% of patients; they are often relatively mild and non-specific, with thickening of interlobular and intralobular septa and parenchymal bands being the most frequently observed [22]. Honeycombing is uncommonly seen in SLE. Other findings include GGO and consolidations, reflecting not only the presence of a wide spectrum of patterns such as interstitial fibrosis, but also the presence of ALP, DAH, or OP [13].

**Fig. 21:**
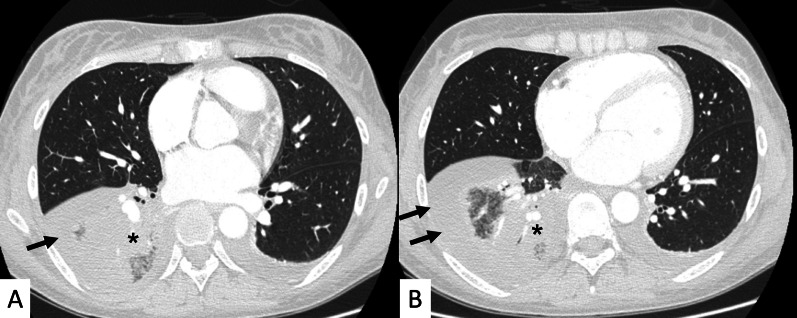
A 37-year-old patient with ALP, characterized by dyspnea, fever and pleuritic pain; on HRCT images (**A**, **B**), bilateral pleural effusions (black arrows) and right lower lobe consolidation (black asterisk) are clearly recognizable

ALP is a rare and fatal form that can be the initial presenting form of SLE; it may be seen concurrently in the context of an active lupus nephritis [66]. It is characterized by a variable degree of respiratory impairment with fever, cough, dyspnea, pleuritic and chest pain; on HRCT, it can be revealed by the presence of focal or diffuse pulmonary consolidations (Fig. 21), often indistinguishable from severe infection and acute respiratory distress syndrome [22]. It is likely that the majority of cases represent DAD and/or DAH [10]. Unfortunately, about 50–100% of patients who survive the acute episode, will progress to chronic interstitial pneumonitis [65].

**Fig. 22:**
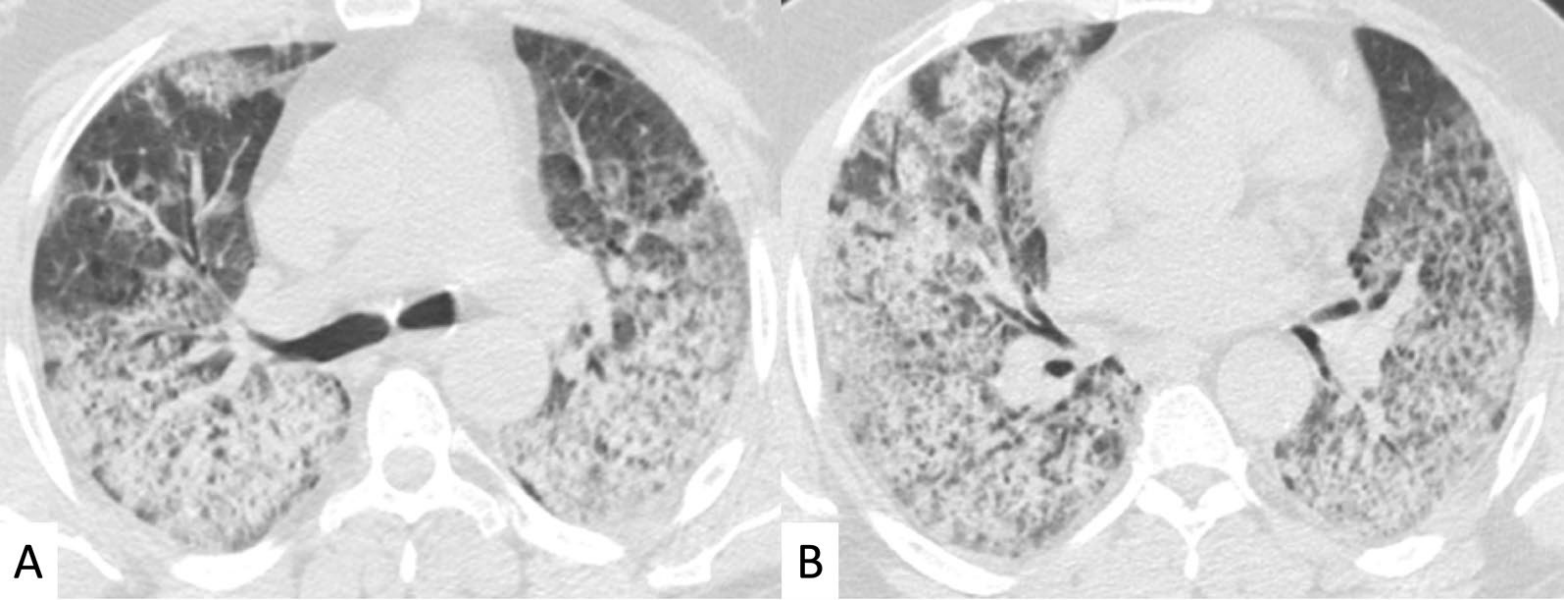
DAH in a patient with LSD. Images (**A**, **B**) show diffuse bilateral consolidations and ground-glass opacities

DAH is a relatively rare but severe lung manifestation (1.5% of SLE patients) (Fig. 22) [10]. Similar to ALP, it is characterized by an abrupt onset of dyspnea, fever and cough, hemoptysis at presentation, with a specific sudden decrease of hemoglobin. HRCT features are comparable to ALP, showing bilateral consolidations and/or ground-glass opacities. BAL is diagnostic, identifying high neutrophil count, serosanguinous fluid, and hemosiderin-laden macrophages within the lavage [70].

Other complications of SLE may include diaphragmatic dysfunction, PH and pulmonary thromboembolism.

Diaphragmatic dysfunction, an uncommon manifestation of SLE, could be related to a reduction of diaphragmatic strength, diaphragm fibrosis, and phrenic nerve palsy. It is manifested by reduced lung volume, reproducing what is known as “the shrinking lung syndrome” [71]. As reported by Mathai et al., risk factors include longer disease duration, presence of anti-RNP antibodies, and history of pleuritis [14]. Patients with shrinking lung syndrome often present dyspnea and chest pain; in these cases, PFTs show a restrictive pattern. Radiographic findings may be aspecific, with elevation of diaphragm and basal atelectasis—but no evidence of ILD or pleural disease [66].

PH occurs in SLE female patients—typically under the age of 40 years; risk factors include the presence of serositis, interstitial disease, Raynaud’s phenomenon, anticardiolipin and anti-U1 RNP antibodies [72]. Dyspnea during exercise is often the first symptom and PFTs may show reduced values; however, the gold standard for diagnosis is right heart catheterization [65]. HRCT is useful to exclude other diseases such as ILD, often showing enlarged pulmonary vessels and compromised pulmonary/aorta ratio.

Pulmonary thromboembolism may be found in those patients with SLE: there is a direct correlation between antiphospholipid (APL) antibodies levels to lupus anticoagulant activity and inhibition of activated protein C, thereby producing a prothrombotic effect and increasing the risk of a thromboembolic event [73]. Clinically, patients may present with sudden onset pleuritic chest pain, dyspnea or hypoxemia. Computed tomography pulmonary angiogram (CTPA) may reveal filling defects of the main arterial pulmonary branches, or segmental and subsegmental pulmonary vessels.

### Sjögren syndrome (SS)

SS, also known as “sicca syndrome”, is a chronic inflammatory autoimmune disease of the exocrine glands, caused by parenchymal lymphocytic infiltration; the disease is characterized by xerostomia and xerophthalmia [74].

This disease is relatively common, and has been found in 0.1% of the population; it has been distinguished in primary or secondary SS, depending on whether it is associated or not with other CTDs—most commonly RA, SLE and SSc [13]. In a retrospective study from the United States, the annual incidence reported for primary SS was 3.9 per 100,000 [75, 76].

A diagnosis of SS is based on the weighted sum of 5 items, which include: (1) labial salivary glands with focal lymphocytic sialadenitis (weight 3 points); (2) identification of anti-nuclear antibodies against ribonucleoproteins Ro/SSA (weight 3 points); (3) ocular staining score ≥ 5 in at least 1 eye (weight 1 point); (4) Schirmer’s test ≤ 5 mm/5 min in at least 1 eye (weight 1 point); (5) unstimulated saliva flow rate ≤ 0.1 ml/min (weight 1 point). According to this ACR-EULAR 2016 Classification Criteria, diagnosis of Sjögren Syndrome is achieved in patients having a score ≥ 4 [77].

Extra-glandular and systemic manifestations involve kidney, central nervous system and lung; the presence of the latter is associated with a fourfold increased risk of mortality after 10 years of the disease [14, 74].

#### Lung involvement

The prevalence of pulmonary involvement in SS ranges from 9 to 75%, depending on inclusion criteria and on the detection method employed [74]. More in detail, the prevalence of clinically significant lung involvement ranges from 9 to 24%, according to the different studies reported in the paper by Mathai et al. [14]; however, about 75% of asymptomatic patients show abnormalities on pulmonary function tests, BAL, and HRCT [78–80]. Pulmonary manifestations in SS are rarely the presenting feature and usually develop late in the course of the disease, similar to RA and SLE [14].

Lung involvement includes a wide spectrum of airways (as follicular bronchiolitis, common findings) and interstitial abnormalities [79, 80], and lymphoproliferative disorders [10, 81].

SS-related ILD is a common manifestation; it is associated with an increase in mortality, which seems to be related to the baseline arterial partial pressure of oxygen, and to the presence of honeycombing on HRCT [74]. According to some studies, smoking, male sex, long disease evolution, systemic manifestations, and the presence of anti-Sjogren’s syndrome-related antigen A (SSA) antibodies (also called anti-Ro or anti-SSA/Ro antibodies) have been identified as main risk factors for lung disease [74].

#### Radiological features

NSIP is the most frequent subtype of ILD in SS, with a prevalence of 28–61% [14]. In the past, early studies have reported LIP as the main form of ILD. However, in a case series published in literature, only 17% of patients with Sjögren have shown a pattern of LIP histologically. This recent change—regarding the most frequent pattern, could be related to the revisions of diagnostic criteria for LIP, with a reclassification as LIP of some cases of cellular NSIP; in addition, others previously classified as LIP, would now be considered as low-grade lymphoma [10, 14].

Histologically, LIP is characterized by a diffuse interstitial and peribronchiolar infiltration of lymphoplasma cells that significantly expands the alveolar septa [81].

After the NSIP and LIP pattern, SS-ILD may reproduce an appearance of OP; UIP, in a minority of cases, may also be observed.

Parenchymal abnormalities are evident at chest radiography in 10–30% of patients, with a reticulonodular pattern that involves mainly the lower lung zones [13].

**Fig. 23:**
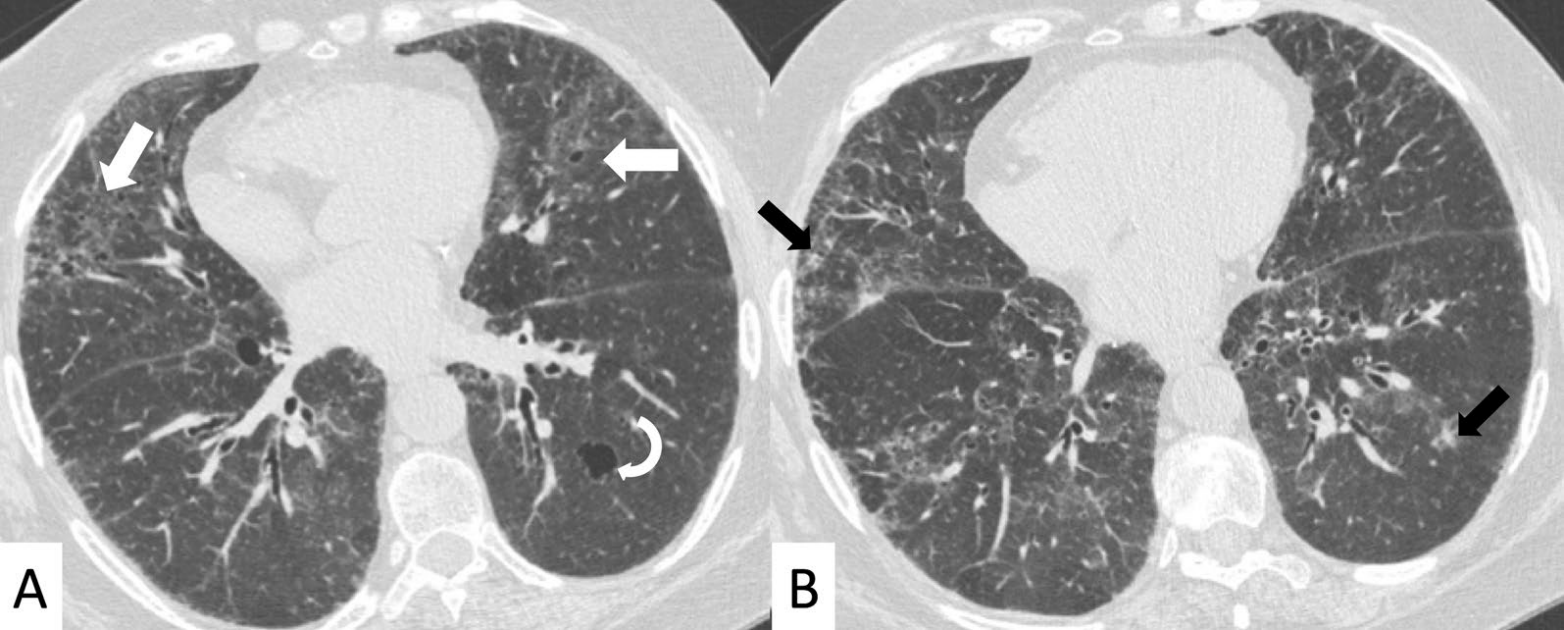
LIP pattern in a 77-year-old female patient. **A**, **B** Ground-glass opacifications superimposed on a reticular pattern (white arrows in **A**)—reflecting the lymphocytic infiltration; there are also thin-walled peribronchovascular cysts (white curved arrow in **A**) and centrilobular and subpleural nodules (black arrows in **B**)

**Fig. 24:**
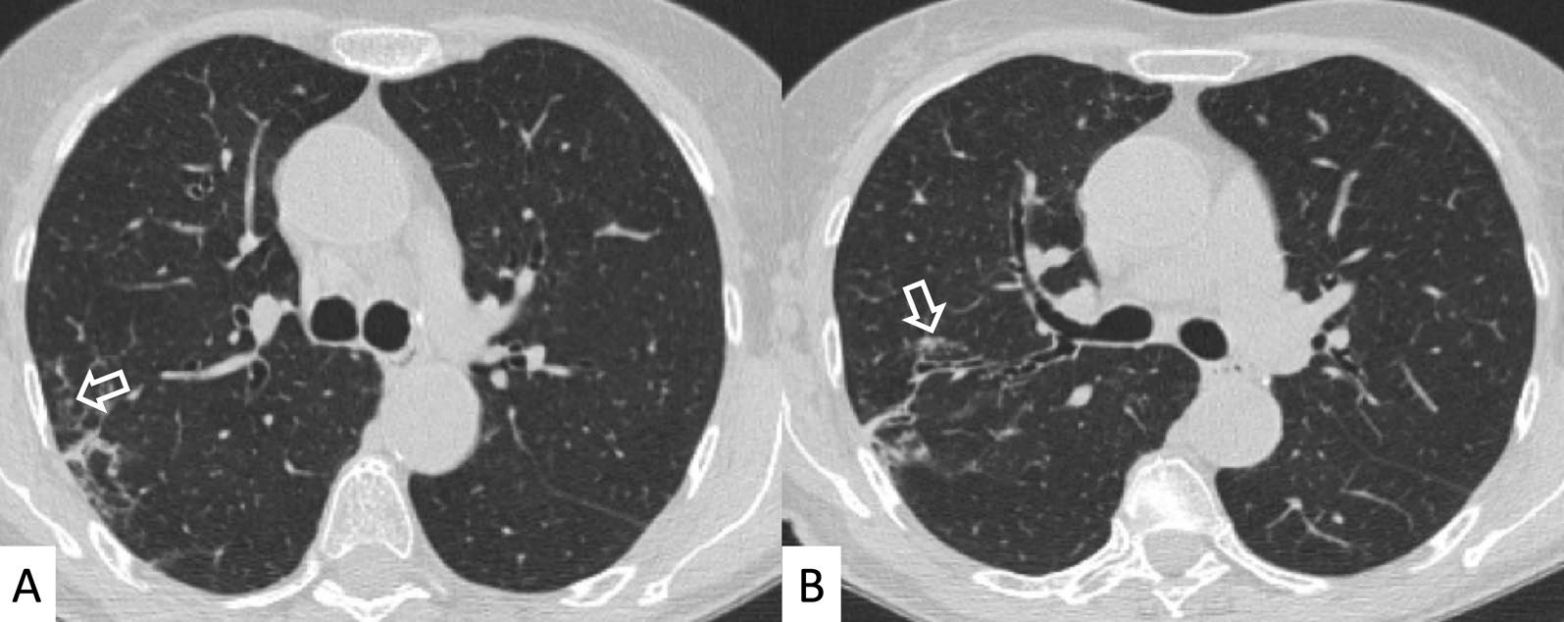
Pulmonary nodules and reticulations in a pattern LIP. Nodules (white empty arrows in **A**, **B**) may be reproduced in LIP patients, due to the presence of lymphocytic bronchiolitis, with thickening of the bronchovascular bundles

**Fig. 25:**
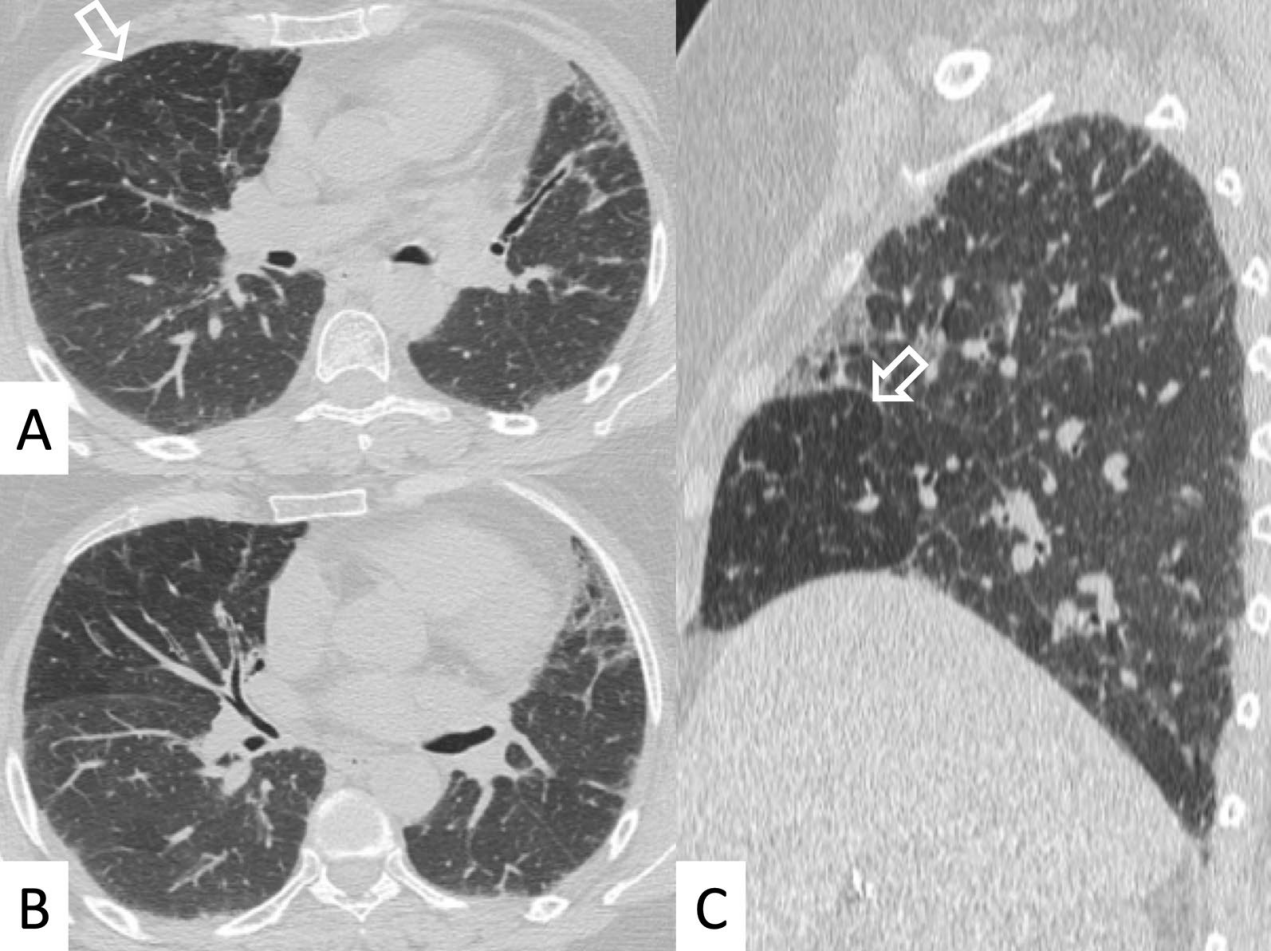
LIP pattern with inhomogeneous attenuation of pulmonary parenchyma. Nodules, due to the peribronchovascular locations, may reproduce inhomogeneous lung attenuation, due to air-trapping mechanisms (white empty arrows in **A**, **C**)

**Fig. 26:**
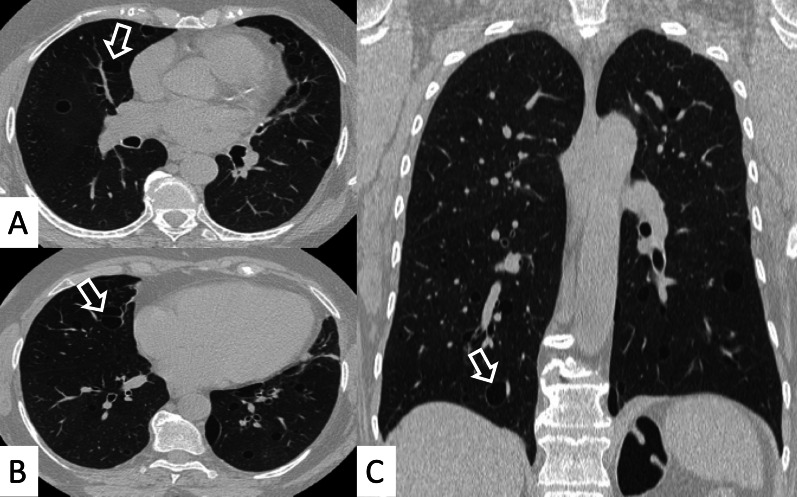
Typical peribronchovascular locations of cysts in a female patient with SS. Cysts—sharply demarcated and thin-walled—are distributed along peribronchovascular structures (white empty arrows in **A**–**C**); they may be not associated with other pulmonary findings

**Fig. 27:**
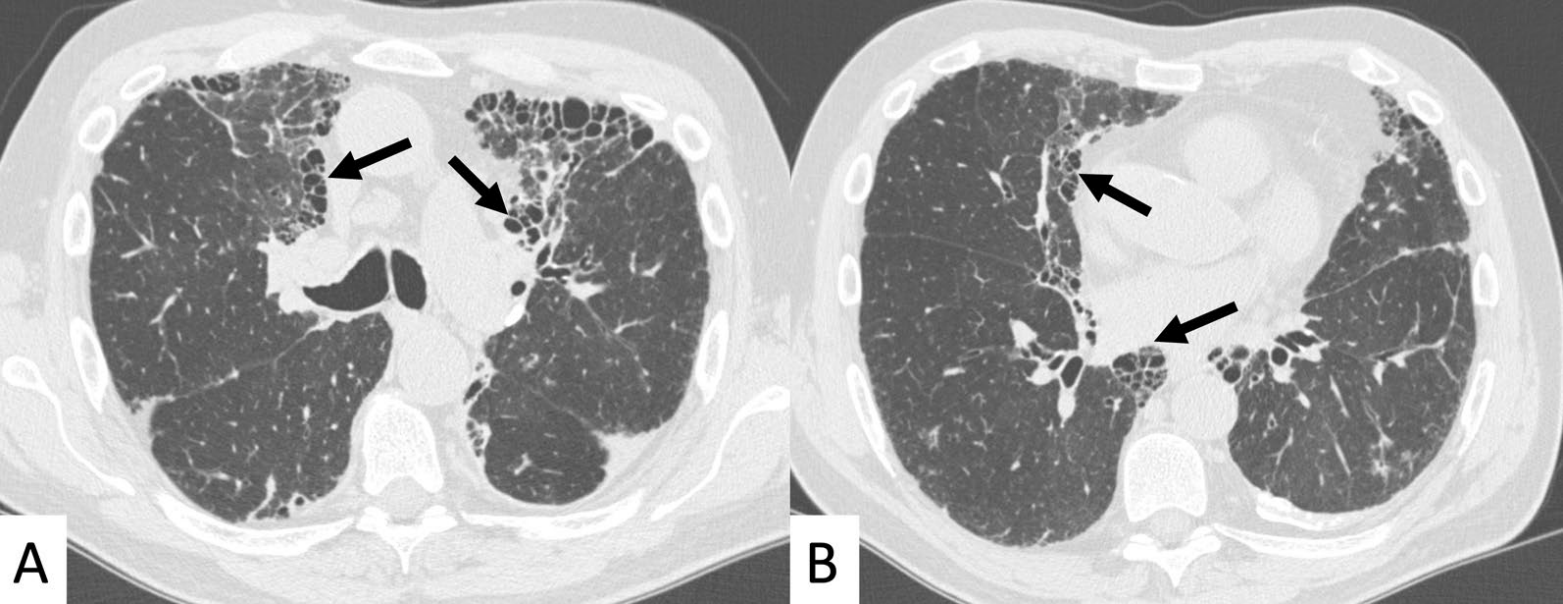
A 77-year-old man with diagnosis of Sjöegren, achieved from histological samples of parotid glands. Cystic spaces are clearly depicted through the lung regions, with anterior and subpleural mediastinal location (black arrows in **A**, **B**). Peripheral cystic areas have been associated with perilymphatic distribution of disease

**Fig. 28:**
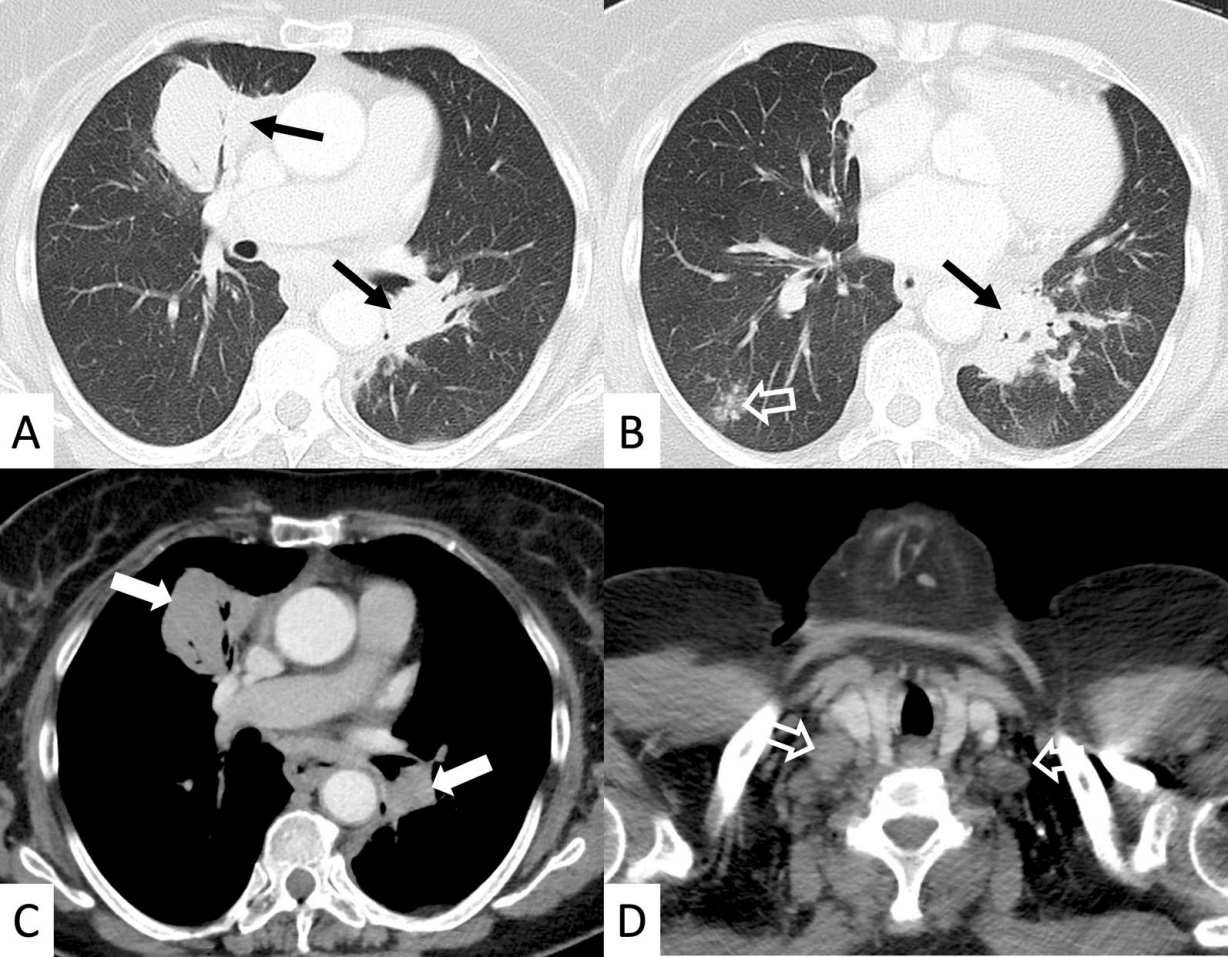
A 77-old-year female patient affected by SS. After a chest radiograph—which showed pulmonary consolidations—she underwent a CT examination. **A**, **B** Right and left pulmonary consolidations are depicted (black arrows in the middle and left lower lobes); **B** a ground-glass attenuation area, with small nodules, is also demonstrated (empty white arrow). On mediastinal windowing level, the consolidations have homogenous density, appearing as soft attenuation masses (white arrows in **C**). A CT scan acquired at the cervical level, clearly demonstrate enlarged nodes in the supraclavicular spaces (white empty arrows in **D**); histological samples confirmed the development of a lymphoma in this SS patient. Consolidations, large nodules (> 1 cm) or pleural effusions; all these signs have been associated with lymphoma

HRCT abnormalities findings have been described in a range of 34–50% of cases; main radiological patterns include ground-glass attenuation areas, reticular opacities and consolidations—while honeycombing occurs infrequently [82]. An HRCT pattern consistent with NSIP has been found to be highly correlated with a histological NSIP pattern, so that a surgical biopsy is not required. In other cases, a typical HRCT pattern of LIP may be observed; it includes GGO—reflecting the lymphocytic infiltration, thin-walled peribronchovascular cysts and centrilobular and subpleural nodules (Fig. 23). The pulmonary nodules (Fig. 24) may be explained by the presence of lymphocytic bronchiolitis; thickening of the bronchovascular bundles is also observed, related to the expansion of the interstitial tissue by lymphoplasma cell infiltration [81]; these nodules, due to the peribronchovascular locations, may determinate inhomogeneous lung attenuation, due to air-trapping mechanisms. (Fig. 25). Cysts may have a typical peribronchovascular or peripheral location, being detected along peribronchovascular bundles (Fig. 26) or subpleural mediastinal regions (Fig. 27). Unfortunately, an LIP pattern is not clearly distinguishable from malignant lymphoma, which occurs approximately in 20% of patients with SS. To achieve a differential diagnosis, radiologists should consider other radiological features—such as consolidations, large nodules (11–30 mm) or pleural effusions; all these signs have been associated with lymphoma (Fig. 28) [83].

### Mixed connective tissue disease (MCTD)

MCTD has been defined “overlap syndrome”, since it is characterized by a mixture of signs and symptoms of SSc, PM and SLE; these clinical conditions may be revealed together, or sequentially during observation [84]. MCTD can affect all ages, with a peak incidence reached around the age of 40 years [85].

The first definition of MCTD as a separate CTD was introduced in 1972 by Sharp [86]; however, some rheumatologists believe that MCTD does not represent an overlap syndrome—but an early and unspecific phase of an evolving, more distinct CTD [19].

Diagnosis of MCTD should be considered in patients having positive anti-U1 RNP antibodies and presenting clinical manifestations such as Raynaud's phenomenon and diffuse hand edema (“puffy hands”); these patients, however, do not meet all the diagnostic criteria of other CTDs (SSc, PM and SLE) [19]. Patients must have at least two of the following clinical features: arthritis, myositis, leukopenia, esophageal dysmotility, pleuritis, pericarditis, ILD or PH [10, 85].

#### Lung involvement

A large number of patients with MCTD have pulmonary involvement; however, in the majority of cases, patients show relatively mild disease, and many are asymptomatic [87, 88].

According to Narula et al., approximately 50% of patients with MCTD develop a radiologic ILD pattern [89]. The presence of dysphagia is associate with ILD in MCTD patients; some case reports have also described associations between Raynaud’s phenomenon and development of ILD [90].

#### Radiological features

In patients with MCTD, the main radiological abnormalities resemble those seen in SLE, SS, and PM; thus, HRCT findings are heterogeneous [90] and most frequent alterations include interstitial pneumonitis and fibrosis (fibrotic NSIP), PH, pleural and pericardial effusion [28].

As in the other CTDs, ILD is associated with an increased risk of mortality [51] and its course is usually SSc-like; the progression is slow in the majority of patients.

Severe fibrosis has been associated with older age, but not with length of diagnosis or smoking. The positivity of anti-Ro52 antibodies can also be related to a severe degree of fibrosis, similarly to what happens in SS-ILD [84].

**Fig. 29:**
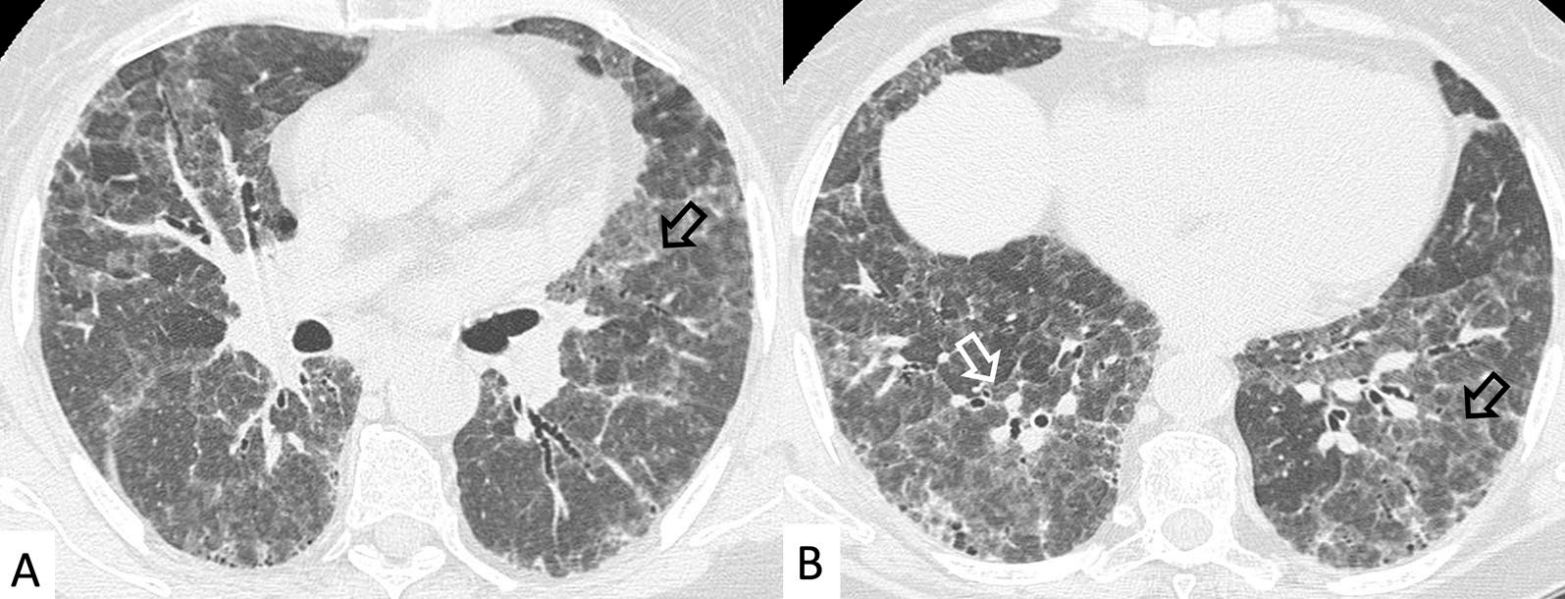
A NSIP pattern in a female patient with diagnosis of MCTD. HRCT show ground-glass areas (white and black empty arrows) in figures **A** and **B**. Reticulations and small cystic areas are also evident, in this fibrosing pattern

The most common radiological pattern is NSIP (Fig. 29), with predominant ground-glass attenuations; UIP is also common as interstitial pattern in patients having MCTD. In a recent case series, interstitial diseases varied from interstitial pneumonitis with bronchiectasis, to more specific NSIP pattern and UIP pattern [89].

**Fig. 30:**
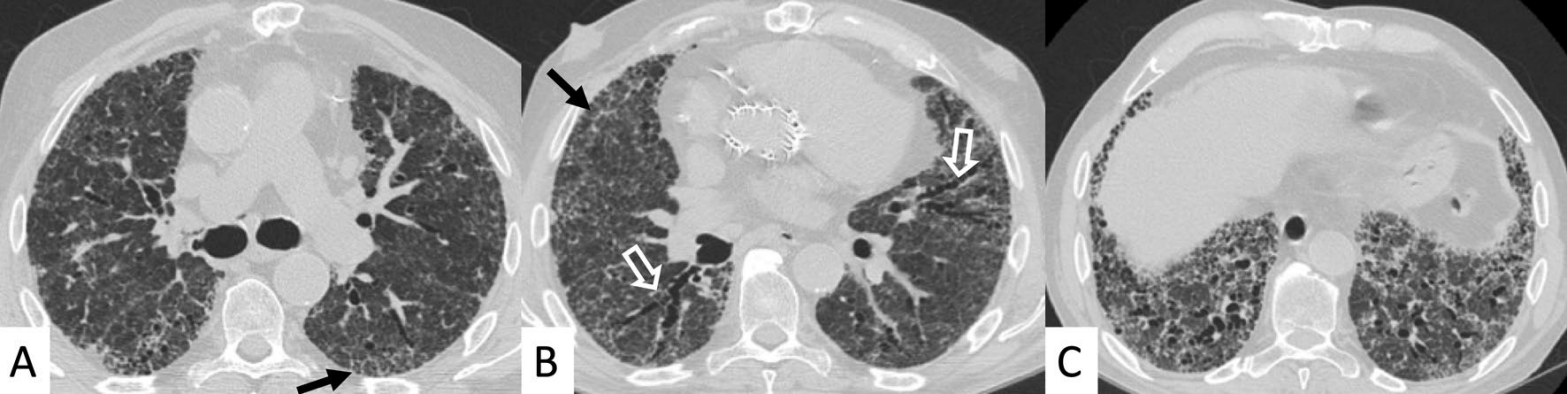
A 70-year-old man with MCTD, positive for ANCA antibodies. HRCT images show reticulations (black arrows in **A, B**) and bronchiectasis, with irregular course through the lung (white empty arrows), and cystic appearance, clearly recognizable in the basal regions in **C**

Reticulations and bronchiectasis—signs of interstitial pneumonitis—are typically reported in these patients (Fig. 30); honeycombing, consolidations, and poorly defined centrilobular nodules are less commonly encountered [22]; in some cases, OP and DAD have been occasionally described in MCTD patients, and may be related to an acute exacerbation of disease [19]. Lastly, other important complications of MCTD include PH, vasculitis, pulmonary thromboembolism and esophageal dysmotility [91].

## Conclusions

Pulmonary manifestations may be heterogeneous in CTDs, and ILDs are not the only alterations which can be found on imaging. All pulmonary compartments—such as airways, interstitium, pulmonary vessels and pleura may be interested, namely in RA, SSc and SS diseases. The heterogeneous lung involvement leads to “a broad spectra of clinical manifestations” [32], which may be not easily recognized by clinicians and radiologists.

Most frequent ILD patterns found on imaging are represented by UIP and NSIP; however, some other radiological scenarios—including OP and LIP—may be found, respectively, in PM/DM or ASSD, or in patients with Sjögren.

Drug induced diseases and/or aspiration pneumonia could be the radiological abnormalities found in PM/DM.

A classification—based on radiological involvement of pulmonary compartments—should be adopted by radiologists: a differentiation between CTDs with and without ILDs, should be considered of crucial significance—since some patterns of ILDs are related to disease severity and disease progression [92]. Among CTD-ILDs, it can be useful to distinguish clearly diseases which may reproduce UIP pattern on HRCT: in several studies, same IPF-like behaviors have been reported in RA or Interstitial Pneumonitis with Autoimmune Features (IPAF) having UIP patterns on imaging, or showing honeycombing on images. In this regard, a future classification based on radiological features—indicating bad worse prognosis or progressive phenotype—is needed.

In some CTDs, radiological abnormalities are not specific—so that a multidisciplinary approach is strongly recommended: a combination of serological markers, clinical findings and radiological features is required to achieve a correct and prompt diagnosis. In addition, research studies are needed to recognize serological and radiological markers—in order to predict disease progression in early phases; in this regard, the identification of progressive radiological abnormalities is mandatory, in order to introduce therapeutic options for patients with progressive phenotype ILDs. In the near future, quantitative CT will probably play an important role, increasing the identification of patients with ILD progression, characterized by increased extent of fibrosis on HRCT not clearly appreciable on radiological images.

## Abbreviations

ACA: Anticentromere antibodies
ACR-EULAR: American College of Rheumatology-European League against Rheumatism
ALP: Acute lupus pneumonitis
ALP: Antiphospholipid
ANA: Antinuclear antibodies
ASSD: Antisynthetase syndrome
BAL: Bronchoalveolar lavage
CCP: Cyclic citrullinated peptide
CK: Creatine kinase
CTDs: Connective tissue diseases
CTPA: Computed tomography pulmonary angiogram
DAD: Diffuse alveolar damage
DAH: Diffuse alveolar hemorrhage
DIP: Desquamative interstitial pneumonia
DLCO: Diffusing capacity of the lungs for carbon monoxide
DM: Dermatomyositis
FVC: Forced vital capacity
GGOs: Ground-glass opacities
HRCT: High resolution computed tomography
IIMs: Idiopathic inflammatory myopathies
ILA: Interstitial lung abnormalities
ILDs: Interstitial lung diseases
IPAF: Interstitial pneumonia with autoimmune features
IPF: Idiopathic pulmonary fibrosis
LIP: Lymphocytic interstitial pneumonia
MCTDs: Mixed connective tissue diseases
NSIP: Non-specific interstitial pneumonia
OP: Organizing pneumonia
PFTs: Pulmonary function tests
PH: Pulmonary hypertension
PM: Polymyositis
RA: Rheumatoid arthritis
RF: Rheumatoid factor
RNP: Ribonucleoprotein
SLE: Lupus erythematosus systemic
SS: Sjögren syndrome
SSA: Sjogren’s syndrome-related antigen
SSc: Systemic sclerosis
UIP: Usual interstitial pneumonia
